# Cerebral amyloid angiopathy: a narrative review

**DOI:** 10.3389/fnagi.2025.1632252

**Published:** 2025-09-16

**Authors:** Natalia Motzko Noto, Robert C. Speth, Lisa S. Robison

**Affiliations:** ^1^Department of Pharmaceutical Sciences, Barry and Judy Silverman College of Pharmacy, Nova Southeastern University, Fort Lauderdale, FL, United States; ^2^Department of Pharmacology and Physiology, School of Medicine, Georgetown University, Washington, DC, United States; ^3^Department of Psychology and Behavioral Neuroscience, College of Psychology, Nova Southeastern University, Fort Lauderdale, FL, United States

**Keywords:** cerebral amyloid angiopathy, amyloid-beta, animal models, treatment, lifestyle

## Abstract

Cerebral amyloid angiopathy (CAA) is a cerebrovascular disorder characterized by the accumulation of amyloid-beta (Aβ) in the walls of cerebral vessels. It is commonly associated with cognitive decline, cerebral hemorrhage, and other neurological pathologies. Despite its prevalence and impact, there are currently no approved treatments for CAA. CAA frequently co-occurs with Alzheimer’s disease (AD), but affected patients are often excluded from anti-amyloid therapies due to increased risks of cerebral edema and hemorrhage, underscoring the urgent need for alternative and safe approaches for treating individuals with CAA. Over the years, various animal models have been developed to investigate the pathophysiology of CAA and evaluate potential treatments. Recent studies have demonstrated that certain repurposed drugs, originally approved for other conditions, show promise for treating CAA. Additionally, it has been shown that positive lifestyle changes may benefit vascular health, reduce amyloid burden and neuroinflammation, and improve cognitive resilience in individuals with CAA. In this review, we summarize the current knowledge on CAA, its relationship with AD, insights from preclinical and clinical studies, and emerging evidence supporting the potential of drug repurposing and lifestyle modification in managing CAA.

## Graphical Abstract

**Figure:**
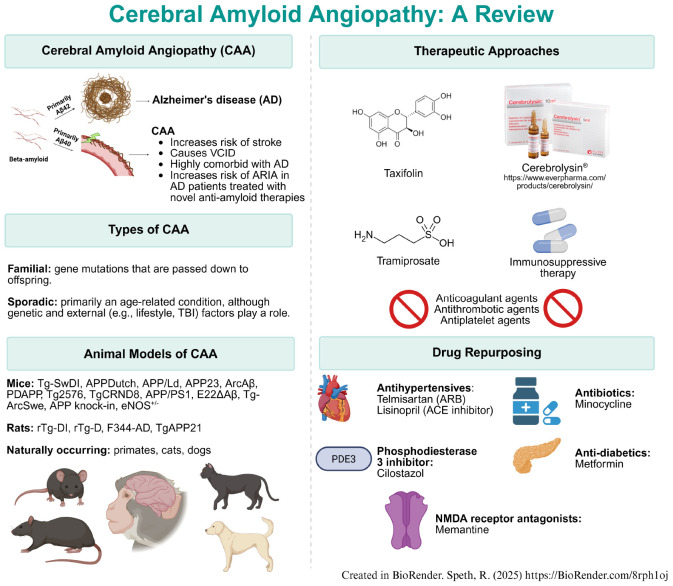
A review of cerebral amyloid angiopathy, including background, differences between familial and sporadic forms of the disease, animal models typically used to study the disease, therapeutic approaches, and drug repurposing potential.

## 1 Introduction

**FIGURE 1:**
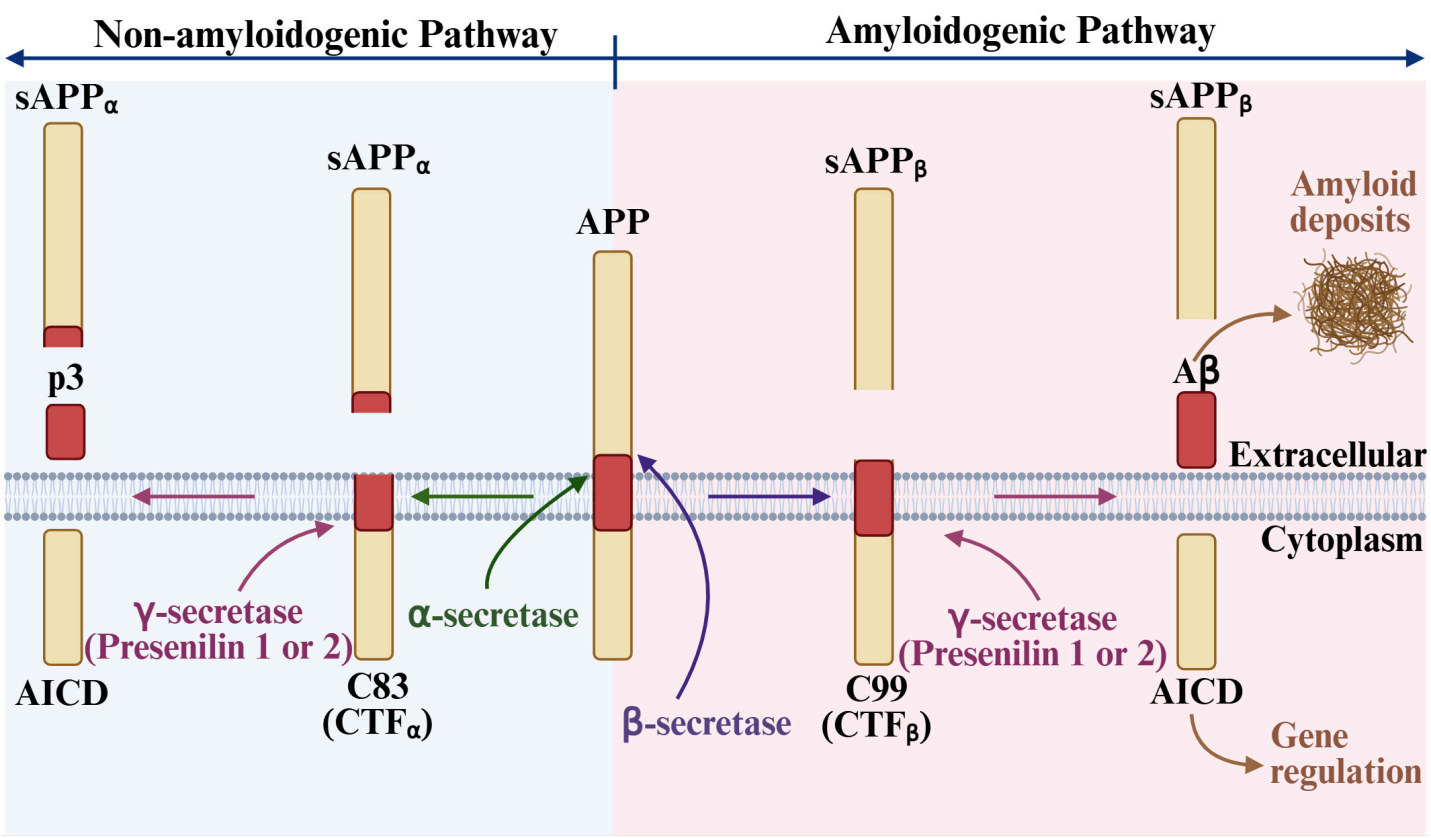
Aβ precursor protein (APP) processing pathways and amyloid-beta (Aβ) formation. APP is processed via two pathways: non-amyloidogenic and amyloidogenic. The amyloidogenic pathway is more heavily associated with neurodegeneration, due to the formation, accumulation, and aggregation of Aβ peptides, while the non-amyloidogenic pathway predominates in healthy individuals (Tsatsanis et al., 2020). Cleavage of APP by β-secretase, gives rise to two substrates: soluble APP_β_ (sAPP_β_) and β C-terminal fragment (CTF_β_) (Selkoe et al., 1996; Wang et al., 2010). The latter is further cleaved by γ-secretase, releasing Aβ peptides that are 39–43 amino acids in length (Wang et al., 2010). Of note, although the amyloidogenic pathway is also present in healthy individuals, Aβ is successfully cleared and does not result in accumulation. Aβ, amyloid-beta peptide; AICD, APP intracellular domain; CTF, C-terminal fragment; p3, p3 peptide; sAPP, soluble APP. Created in BioRender. Speth, R. (2025) https://BioRender.com/w68c366.

Cerebral amyloid angiopathy (CAA) is a common cerebrovascular disorder characterized by the deposition and accumulation of amyloid-beta (Aβ) peptide aggregates in the walls of small- to medium-sized arteries, arterioles, and capillaries of the leptomeninges and cerebral cortex (Weller et al., 2008; Biffi and Greenberg, 2011; Mendel et al., 2013; DeSimone et al., 2017; Chwalisz, 2021). The Aβ peptide is formed through cleavage of the Aβ precursor protein (APP), a neuronal transmembrane glycoprotein (Szidonya and Nickerson, 2023), via the amyloidogenic pathway (Selkoe et al., 1996; Wang et al., 2010; Figure 1). As Aβ aggregates accumulate, they progressively destroy and replace the vascular smooth muscle cells in the tunica media of cerebral blood vessels. Over time, Aβ can infiltrate other vessel wall layers and compromise the structural integrity of the vessel, ultimately leading to rupture and hemorrhage (Weller et al., 2008; Biffi and Greenberg, 2011; Mendel et al., 2013; Greenberg et al., 2020). Reflecting the distribution of Aβ deposition, hemorrhages most often occur in cortical vessels (Kuhn and Sharman, 2025).

Despite being a significant contributor to cognitive decline and the second leading cause of spontaneous intracerebral hemorrhage (ICH) in the elderly after hypertension, CAA currently has no cure or effective treatments. The clinical urgency is further highlighted by the fact that as of August 11th, 2025, there are 10 clinical trials for CAA actively recruiting participants, yet none of its subtypes have approved therapies.

The body of research on CAA is substantial and growing. A PubMed search of “cerebral amyloid angiopathy” yields 4,611 results as of August 11th, 2025, dating back to 1954. Current review papers on CAA often provide a broad overview of existing knowledge. While they summarize findings, many fail to critically address the significant methodological differences across studies, making it difficult to draw reliable conclusions and compare results. A common issue is the lack of a clear strategy for translating findings from animal models to human patients, which is a crucial step for developing effective therapies. The field is evolving rapidly, with new insights into its relationship with Alzheimer’s disease, the potential of drug repurposing, and the impact of lifestyle factors emerging continuously. This review aims to fill that gap by providing a consolidated overview of the current state of knowledge in these areas.

In this paper, we review CAA pathophysiology, prevalence, and diagnostic techniques. We further delve into contemporary therapeutic approaches, the utility of various animal models, the influence of external factors on CAA risk and progression, and the promising strategy of drug repurposing for its management. By synthesizing preclinical findings, clinical trial data, and observational studies, this review offers a thorough and current perspective on CAA, intended to be a valuable resource for clinicians and researchers.

## 2 Methods

To ensure a high-quality review of the literature, a comprehensive search of PubMed and Google Scholar was conducted from August 2024 to August 2025, using key terms such as “cerebral amyloid angiopathy,” “cerebral amyloid angiopathy diagnosis,” “cerebral amyloid angiopathy biomarkers,” “cerebral amyloid angiopathy prevalence,” “cerebral amyloid angiopathy subtypes,” “animal models of cerebral amyloid angiopathy,” “treatment of cerebral amyloid angiopathy,” “cerebral amyloid angiopathy and Alzheimer’s disease,” “cerebral amyloid angiopathy and lifestyle,” “drug repurposing for cerebral amyloid angiopathy,” “taxifolin and cerebral amyloid angiopathy,” “cerebrolysin and cerebral amyloid angiopathy,” “tramiprosate and cerebral amyloid angiopathy,” “cerebral amyloid angiopathy-related inflammation,” “diet and cerebral amyloid angiopathy,” “Mediterranean diet and cerebral amyloid angiopathy,” “hypertension and cerebral amyloid angiopathy,” “exercise and cerebral amyloid angiopathy,” “stress and cerebral amyloid angiopathy,” “sleep and cerebral amyloid angiopathy,” “smoking and cerebral amyloid angiopathy,” “alcohol and cerebral amyloid angiopathy,” and “traumatic brain injury and cerebral amyloid angiopathy.” The search included review articles, case studies, as well as pre-clinical and clinical trials. A total of 317 articles were included in this review to cover the topics of interest. Abstracts, lectures, and newspaper articles were excluded. Each article was carefully assessed to correctly capture relevant information and numerical data. Work cited in relevant papers was taken into consideration in order to properly cite original work. Additionally, ongoing clinical trials worldwide have been assessed^1^ and included in this review, excluding those that have been withdrawn, terminated, or currently have unknown status.

## 3 CAA

### 3.1 Overview and subtypes

The severity of CAA correlates with the extent of pathological changes in cerebral blood vessels. In healthy blood vessels, the Aβ peptide, along with soluble metabolites and interstitial fluid, are drained between the smooth muscle cells in the tunica media and are eliminated through a process called perivascular clearance; while in a CAA-affected brain, this mechanism is impaired, leading to Aβ aggregation, gradual destruction of smooth muscle cells, and destruction of the vessel wall, resulting in brain hemorrhage (Weller et al., 2008; Greenberg et al., 2020). In mild CAA, Aβ is restricted to the tunica media, without significant destruction of smooth muscle cells. In moderate CAA, Aβ begins to replace the tunica media, and the vessel wall becomes abnormally thickened. Severe CAA is characterized by extensive Aβ deposition, fibrinoid necrosis, microaneurysm formation, and blood leakage through compromised vessel walls (Table 1; Vonsattel et al., 1991; Biffi and Greenberg, 2011). After hypertension, CAA is the second most common cause of spontaneous intracerebral hemorrhage (ICH) in people over the age of 60 (Szidonya and Nickerson, 2023) and, when it occurs, episodes are recurrent and typically more severe than the previous one (Passero et al., 1995). Secondary to cerebral hemorrhage and infarcts, CAA can result in cognitive impairments, such as deficits in perceptual speed and episodic memory, and dementia (Chwalisz, 2021; Kozberg et al., 2021).

**TABLE 1:** Cerebral amyloid angiopathy (CAA) severity (Weller et al., 2008; Greenberg et al., 2020).

Parameter analyzed	Mild CAA	Moderate CAA	Severe CAA
Aβ accumulation	Mild	Moderate	Severe
Smooth muscle cells in the tunica media of blood vessels	Intact	Partially destroyed	Fully destroyed
Blood vessel wall	Intact	Intact	Continuous CAA progression may lead to destruction, resulting in brain hemorrhage

Cerebral amyloid angiopathy is classified as either type I or II, based on the predominant vascular compartment affected by Aβ (Thal et al., 2002). In CAA type I, Aβ accumulates mainly in cortical capillaries, as well as leptomeningeal and cortical arteries, arterioles, veins, and venules (Thal et al., 2002; Rajpoot et al., 2022). In contrast, CAA type II, the more common type in sporadic cases (Walker et al., 2024), is characterized by Aβ deposition in the walls of larger leptomeningeal and cortical vessels, sparing the capillaries. Type II is associated with increased formation of fibrils and recurrent hemorrhages, but typically not with brain atrophy or neurofibrillary tangle formation (Timmers et al., 1990; Wisniewski et al., 1991; Wattendorff et al., 1995).

Although CAA is typically considered non-inflammatory, perivascular inflammation may occur with aging due to high levels of Aβ, contributing to loss of vascular integrity and function (Miao et al., 2005; Park et al., 2014; Rosas-Hernandez et al., 2020; Chwalisz, 2021; Kozberg et al., 2021, 2022). When Aβ accumulates in the capillaries (CAA type I), the risk of inflammation increases, sometimes giving rise to a distinct inflammatory subtype called CAA-related inflammation (CAA-ri) (Grasso et al., 2021). An additional subtyping classification is CAA with and without deep perforator arteriopathy (DPA) (Goeldlin et al., 2024). DPA, like CAA, is a common form of sporadic cerebral small vessel disease that affects small arteries, veins, arterioles, venules, and capillaries in deep supratentorial structures (such as basal ganglia and thalamus) and in the brainstem (Schreiber et al., 2020; Goeldlin et al., 2023). Both conditions share several similarities, such as being more prevalent in the aging brain, being major causes of ischemic stroke and ICH, and leading to cognitive decline (Schreiber et al., 2020). However, while in CAA the alterations in brain network are more predominant in the occipital and posterior temporal lobes, in DPA those are more widely seen in the frontal and lateral temporal lobes (Schreiber et al., 2020). Similarities and differences suggest that CAA and DPA, although separate conditions, can also coexist (Schreiber et al., 2020).

The majority of CAA cases are sporadic, primarily associated with aging, and influenced by a combination of genetic susceptibility and environmental and lifestyle factors; however, familial forms of CAA do exist. Familial CAA has been linked to rare autosomal dominant mutations, many of which affect the amyloid precursor protein (APP) gene. The Dutch (E693Q) and Iowa (D694N) mutations in the APP gene result in the loss of neighboring negatively charged residues in the Aβ peptide, promoting its aggregation and likelihood for vascular deposition over parenchymal plaque formation (Van Nostrand et al., 2001; Davis et al., 2004; Baumketner et al., 2008). The Iowa mutation, associated with CAA type I (Rajpoot et al., 2022), is classified as a non-hemorrhagic variant but is highly toxic to cerebrovascular smooth muscle cells. It induces extensive structural changes and promotes perivascular inflammation, involving activation of astrocytes and microglia (Shin et al., 2002). Conversely, the Dutch mutation is associated with CAA type II (Rajpoot et al., 2022). Other APP mutations that give rise to familial CAA include the Icelandic, British, Danish, and Familial amyloidosis-Finnish mutations (Vargas-George and Dave, 2022). Additionally, the Italian (E693K) and Piedmont (L705V) mutations exhibit features of CAA; Arctic (E693G) mutation exhibits features of both CAA and AD; and Flemish (A692G) mutation exhibits vascular Aβ deposition besides parenchymal deposition (Vargas-George and Dave, 2022). In humans, CSF analysis has shown that, in individuals with heritable CAA, decreased Aβ_40_ levels in the CSF is an early feature of CAA pathogenesis (van Etten et al., 2017), likely reflecting its increased sequestration in the cerebral vasculature rather than clearance into the CSF. Similarly, other rare hereditary forms of CAA exist due to mutations in different genes, such as CST3 (or ACYS), PRNP, ITM2B (or BRI2), GSN (or NCBI), TGFBR1, PSEN1, A1AC, MME, LRP1, ACE, variant of CAA and CR1 (Yamada, 2015; Young et al., 2021; Cozza et al., 2023). Mutations in these genes also result in Aβ accumulation, impaired perivascular clearance, and disruption of cerebral blood vessels (Cozza et al., 2023). These findings highlight the distinct pathophysiological features of familial CAA and its relevance to sporadic forms.

Different alleles of the apolipoprotein E (ApoE), a protein involved in lipid transport, fat metabolism, and brain injury repair (Liu et al., 2013), are recognized as important polygenic risk factors for both Alzheimer’s disease (AD) and CAA (DeSimone et al., 2017). The APOE ε4 allele is associated with a dose-dependent increase in vascular Aβ deposition, cognitive decline, and non-hemorrhagic MRI markers of small vessel disease, such as white matter hyperintensities and perivascular spaces. In contrast, the APOE ε2 allele is associated with a higher risk of lobar ICH and greater cortical superficial siderosis severity in CAA (DeSimone et al., 2017; Charidimou et al., 2019; Voigt et al., 2024). Additionally, the distribution of APOE alleles differs between CAA subtypes: the APOE ε4 allele is over four times more frequent in CAA type 1 (with capillary involvement) than in CAA type 2 (which spares capillaries), suggesting that ε4 may preferentially promote capillary Aβ deposition (Thal et al., 2002). Additionally, exosome sequencing studies suggest that rare genetic variants, such as SORL1, TREM2, ABCA7, and ATP8B4, may also be shared genetic risk factors for both CAA and AD (Grangeon et al., 2024).

Iatrogenic CAA (i-CAA) is a rare form of CAA that typically develops as a late consequence (2–3 decades) of neurosurgical interventions in childhood that causes prion-like spread of misfolded Aβ from cadaveric materials and instruments used in the procedure (Banerjee et al., 2022; Greenberg and Charidimou, 2023; Kaushik et al., 2023; Storti et al., 2023). Several preclinical studies have demonstrated the prion-like properties of Aβ (Kane et al., 2000; Meyer-Luehmann et al., 2006; Stöhr et al., 2012; Burwinkel et al., 2018). Three-months-old Tg2576 mice, unilaterally infused into the hippocampus with dilute supernatants of neocortical homogenates from autopsied AD patients, developed profuse Aβ plaques and vascular deposits in the injected hemisphere 5 months after the injection, showing that Aβ can be seeded *in vivo* (Kane et al., 2000). Similarly, young, male APP23 transgenic mice developed robust Aβ deposition in the hippocampus 4 months after the region was injected with brain homogenates from either autopsied AD patients or from aged, Aβ-laden APP23 mice (Meyer-Luehmann et al., 2006). The same strain of mice, when inoculated in the right cerebral hemisphere with brain homogenates from aged transgenic APP23 mice, sustained an increase in the bioluminescence imaging (which can monitor Aβ and PrP prion kinetics) signal in the brain approximately 261 days post inoculation, with significantly higher Aβ levels after 330 days (Stöhr et al., 2012). Additionally, 6–8-week-old APP/PS1 mice, intracerebrally injected with 10% brain homogenates derived from aged APP/PS1 mice, showed pronounced Aβ plaques and vascular deposition in the thalamus after 360 days. Similar results were observed when mice were intravenously injected with the diluted brain extracts (Burwinkel et al., 2018). Taken together, these results suggest that Aβ, through its seeding properties, can lead to development of CAA (due to Aβ deposition) and progression of CAA (due to Aβ aggregation), in addition to having a potential for propagation, spreading, and transmission. Due to its rarity, the exact prevalence of i-CAA is unknown, although Kaushik et al. (2023) reported 49 through 2023. i-CAA is associated with early onset of disease, though radiological and clinical features, including ICH, seizures, and cognitive impairment, are comparable to sporadic CAA (sCAA) (Banerjee et al., 2022). A recent study reported that CSF and plasma biomarkers for Aβ_40_, Aβ_42_, and total tau were similar between patients with i-CAA and sCAA; however, cognitive dysfunction and cardiovascular risk factors were more prevalent in those with sCAA than i-CAA (Pollaci et al., 2024).

### 3.2 Prevalence

Studies have shown that CAA incidence increases with age. A 1983 postmortem study (*N* = 84, 60 ≤ age ≤ 97) found that CAA was present in 36% of the brains of patients over the age of 60, and in 46% of those over the age of 70 (Vinters and Gilbert, 1983). Similarly, a 2021 meta-analysis of 170 studies including over 73,000 subjects studied post-mortem estimated that approximately a quarter of the study’s general population (individuals who represent the general elderly society, including those with known cerebrovascular or neurodegenerative diseases) presented moderate to severe CAA pathology (mean age = 84.9 years) (Jäkel et al., 2022). Additionally, CAA pathology was found in ∼5% of cognitively normal elderly, 20%–25% of patients with ICH, and 50%–60% of patients with lobar ICH (Jäkel et al., 2022).

Studies have also compared the incidence in men vs. women, although data remain limited compared to other conditions like AD. Between the years of 1971 and 1983 in Japan, 400 brains of individuals who were 40+ years old were age-matched and it was found that CAA was present in 18.3% of the men compared to 28% of the women (Masuda et al., 1988). However, more recent studies have not reported significant differences in CAA incidence in men vs. women (Chwalisz, 2021; Wagner et al., 2021; Koemans et al., 2023), although earlier onset and increased severity were reported in men (Shinohara et al., 2016). A study found that men with sporadic CAA experience earlier onset of symptomatic CAA-related ICH and more lobar microbleeds than women (Koemans et al., 2023). Additionally, men with familial CAA (Dutch-type) had a greater number of symptomatic ICH and a shorter interval between the first and second symptomatic ICH (Koemans et al., 2023). While the influence of sex hormones, particularly the decline in estrogen after menopause, has been hypothesized to affect amyloid processing (Xu et al., 2006; Liang et al., 2010), its direct role in CAA development remains unclear and warrants additional investigation. Notably, there is a lack of sex-stratified analyses in many studies on CAA, necessary to understand CAA-related risk and to evaluate the role of risk factors and efficacy of novel therapeutics. Moreover, data is even more limited for transgender or non-binary individuals and their risk profiles.

Sex differences in CAA have also been explored in mouse models. In Tg-SwDI mice, females have more pronounced deficits in spatial and contextual memory, a higher burden of cerebral microbleeds, lower levels of proinflammatory cytokines IL-1α, IL-2, IL-9, and INF-γ, and higher levels of monocytes/macrophages (Maniskas et al., 2021; Finger et al., 2022; Setti et al., 2022). Similarly, compared to males, 12-months old APP/PS1 female mice exhibit more severe CAA, in addition to higher parenchymal Aβ burden (especially in the hippocampus), astrocytosis and microgliosis, more microhemorrhages, higher levels of phosphorylated tau protein and proinflammatory cytokines, and greater neuronal and synaptic degeneration (Jiao et al., 2016).

Gene by sex interactions have also been observed for CAA-related outcomes. When the effect of ApoE is considered, opposite results are reported in humans and mice. In humans with mild cognitive impairment and AD, the APOE ε4 allele was associated with higher risk of cerebral cortex microbleeds in men than in women, although that was also dependent on other risk factors, such as hypertension, diabetes, and age (Shams et al., 2015; Cacciottolo et al., 2016; Finch and Shams, 2016). However, in E4FAD mice, females showed higher levels of CAA, cortical microbleeds, plaques, and soluble Aβ (Cacciottolo et al., 2016; Finch and Shams, 2016). Further investigation is necessary to explain this discrepancy.

Racial and ethnic differences in CAA and related outcomes have also been explored. An autopsy study of AD patients found similar rates of CAA in Caucasian and African American individuals; this lack of difference was confirmed in an independent sample from the National Alzheimer’s Coordinating Center database (Kamara et al., 2018). Data obtained from the Uniform Data Set suggested that being Hispanic predicted a higher likelihood of severe CAA (Ringman et al., 2014). Yet, another study reported that minority survivors of CAA-related ICH exhibited lower CAA burden as measured on MRI (Castello et al., 2021), while a meta-analysis found that APOE ε4 increased the risk for recurrent lobar ICH in White but not minority survivors of ICH (Marini et al., 2019). These findings may have been confounded by differences in survival outcomes in individuals with similar CAA burden. Some studies have reported differences in regional CAA distribution, lower prevalence and decreased severity of CAA pathology, lower proportion of CAA-related ICH, and lower prevalence of strictly lobar microbleeds in East-Asian populations compared to Western populations (Masuda et al., 1988; Ng et al., 1991; Chen et al., 2010; Yakushiji et al., 2020; De Kort et al., 2024).

There may also be interactions between race/ethnicity and genetic risk factors, like APOE alleles. For example, a meta-analysis reported that APOE ε2 and APOE ε4 were associated with lobar ICH risk in White but not Black or Hispanic participants; however, after controlling for hypertension, APOE ε4 was associated with lobar ICH risk in Hispanic but not Black participants (Marini et al., 2019). Despite these differences, current research is hampered by underrepresentation of non-white populations and reliance on autopsy or referral-based cohorts, which may not be representative. Better population-based data with diverse cohorts is necessary to understand CAA-related risk and to evaluate the role of risk factors and efficacy of novel therapeutics.

### 3.3 Diagnosis and biomarkers

**FIGURE 2:**
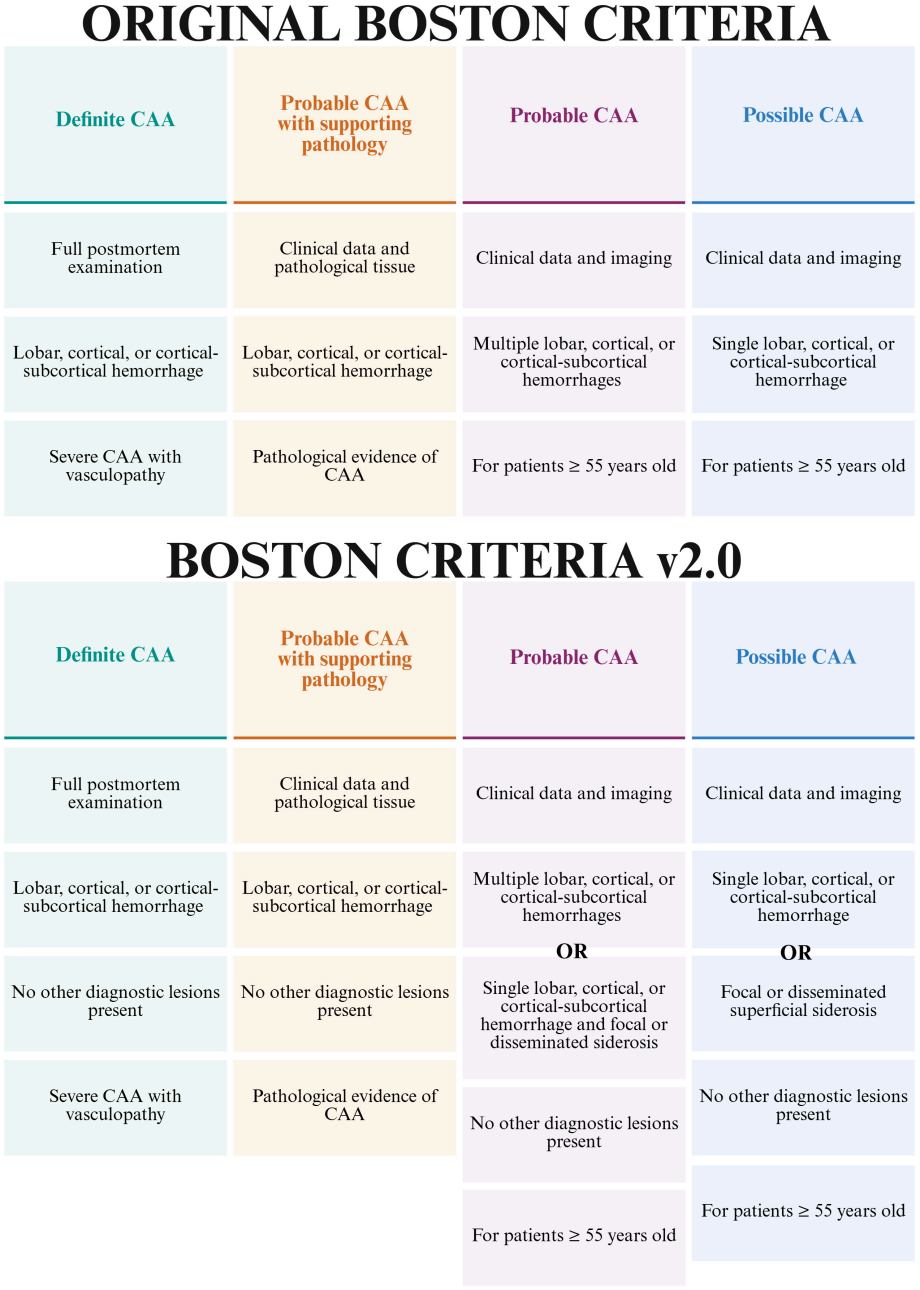
Comparison of original and latest versions of the Boston criteria. Both versions have been previously validated. In a study by Zanon Zotin et al. (2024), 134 individuals were analyzed and, while the original version yielded a sensitivity of 26.5% and a specificity of 90.6%, v2.0 yielded a sensitivity of 38.8% and a specificity of 83.5%. Created in BioRender. Speth, R. (2025) https://BioRender.com/z44kj4a.

A definitive diagnosis of CAA can only be made postmortem through histopathological confirmation of Aβ deposition in cerebral vessel walls (Kuhn and Sharman, 2025). However, clinical diagnostic criteria, most notably the Boston Criteria, are used to diagnose probable or possible CAA in living patients. First introduced in 1995 and recently updated (Figure 2), the latest version (Boston Criteria v2.0), offers improved sensitivity but slightly reduced specificity compared to previous versions (Zanon Zotin et al., 2024). The criteria classify CAA into four categories: definite CAA, probable CAA with supporting pathology, probable CAA, and possible CAA (Illsley and Ramadan, 2014; Kuhn and Sharman, 2025).

Imaging techniques used to diagnose CAA include magnetic resonance imaging (MRI) and computed tomography (CT) scans, which help detect areas of cerebral hemorrhage, most often in the posterior lobar cortical and subcortical regions (Sakurai et al., 2014; Kuhn and Sharman, 2025). In cases where CAA-ri is suspected, a brain biopsy can be performed to confirm the presence of Aβ deposition (Kuhn and Sharman, 2025). Among MRI modalities, T2*-weighted gradient-recalled echo (T2*-GRE) and susceptibility weighted imaging (SWI) are particularly useful for identifying cerebral microbleeds, a hallmark of CAA. SWI is significantly more sensitive than T2*-GRE in detecting these microhemorrhages, with studies reporting that T2*-GRE can miss microhemorrhages in ∼25% of patients (Haacke et al., 2007; Charidimou et al., 2013; Sakurai et al., 2014). Although CT scans can identify larger hemorrhages, they have a lower contrast resolution compared to MRI and are considerably less sensitive for the microhemorrhages seen with SWI MRI (Sakurai et al., 2014; Azmin et al., 2015). Instead, MRI, especially using SWI sequences, remains the preferred modality for detecting the subtle hemorrhagic changes characteristic of CAA. A proposed study using high frequency serial magnetic resonance imaging to prognose CAA (NCT06128824) will attempt to visualize cortical superficial siderosis (cSS) and hemorrhagic and ischemic lesions as surrogate markers for CAA.

Tissue biopsy can be an important tool for diagnosing CAA in life. A study by Greenberg and Vonsattel (1997) examined brain specimens from individuals with confirmed CAA-related hemorrhage and found vascular Aβ deposition in 100% of the specimens with hemorrhage, suggesting high sensitivity under ideal sampling conditions. However, several issues arise from generalizing these results to clinical situations, as the characteristics of biopsied tissues (e.g., size and location) can affect detection of vascular Aβ (Greenberg and Vonsattel, 1997). The distribution of CAA pathology is patchy and segmental, meaning that not all tissue samples will capture affected vessels. This heterogeneity can lead to false negatives if vascular Aβ is absent in the sampled area, which is limited to just a small section of cortical tissue (Greenberg and Vonsattel, 1997). The prevalence of Aβ pathology increases with age, and some degree of vascular Aβ deposition is often present in older individuals; however, its presence does not necessarily indicate that CAA caused a hemorrhage, potentially leading to false positives (Greenberg and Vonsattel, 1997). Therefore, the specificity of biopsy findings is assumed to be age-dependent. In younger patients, the detection of vascular Aβ is more likely to indicate pathological CAA, whereas in older individuals, incidental Aβ deposition may reduce specificity. As such, clinical interpretation of biopsy results must consider both patient age and the inherent limitations of localized sampling (Greenberg and Vonsattel, 1997).

Amyloid-Positron Emission Tomography (PET) scans is a commonly used technique to assess Aβ levels in the brain (Verbeek et al., 2009). Amyloid PET imaging using tracers such as [^11^C]PiB or [^18^F]florbetapir can detect fibrillar Aβ deposits in the brain and is commonly used to assess amyloid pathology in AD (Landau et al., 2013), while ^18*F*^Flutemetamol can be used to help reclassify mixed arteriosclerosis + CAA into CAA pathology and arteriolosclerosis-predominant pathology (Romoli et al., 2024). While CAA can also be visualized by amyloid PET, it is not specific to CAA, as it detects total amyloid burden without distinguishing between vascular and parenchymal deposits. As a result, while a positive amyloid PET scan may suggest the presence of CAA, especially when combined with clinical criteria and MRI findings, it cannot definitively differentiate CAA from AD or confirm a diagnosis of CAA on its own.

Similar to amyloid PET imaging, cerebral spinal fluid (CSF) samples also reflect Aβ levels in the brain. It has been reported that the levels of Aβ_40_ in the CSF are decreased in patients with CAA (Verbeek et al., 2009; Charidimou et al., 2018; Sembill et al., 2023). Although studies have suggested that the levels of Aβ_40_ in the CSF are significantly lower in CAA compared to AD, CSF analysis alone is insufficient to fully differentiate between the two conditions (Verbeek et al., 2009; Charidimou et al., 2018; Sembill et al., 2023). A study by Sembill et al. (2023) identified a distinct CSF biomarker profile in CAA patients compared to patients with AD, mild cognitive impairment due to AD, mild cognitive impairment with unlikely AD, and healthy controls (Table 2).

**TABLE 2:** Cerebral spinal fluid (CSF) pattern in cerebral amyloid angiopathy (CAA) patients according to Sembill et al. (2023).

Parameter analyzed	CAA vs. AD	CAA vs. mild cognitive impairment due to AD	CAA vs. mild cognitive impairment with unlikely AD	CAA vs. healthy controls
Aβ_40_	↓	↓	↓	↓
Aβ_42_	Comparable	Comparable	↓	↓
Tau protein	↓	↓	↑	↑

↓, Reduced parameter in CAA; ↑, Increased parameter in CAA.

In parallel with the development of blood biomarkers for AD, similar studies are in progress to prognose and diagnose CAA (NCT06960538). These biomarkers include Aβ_40_, Aβ_42_, total tau (t-tau), neurofilament light chain (NfL), and glial fibrillary acidic protein (GFAP), in addition to lipidomic profiling.

### 3.4 CAA and AD

**FIGURE 3:**
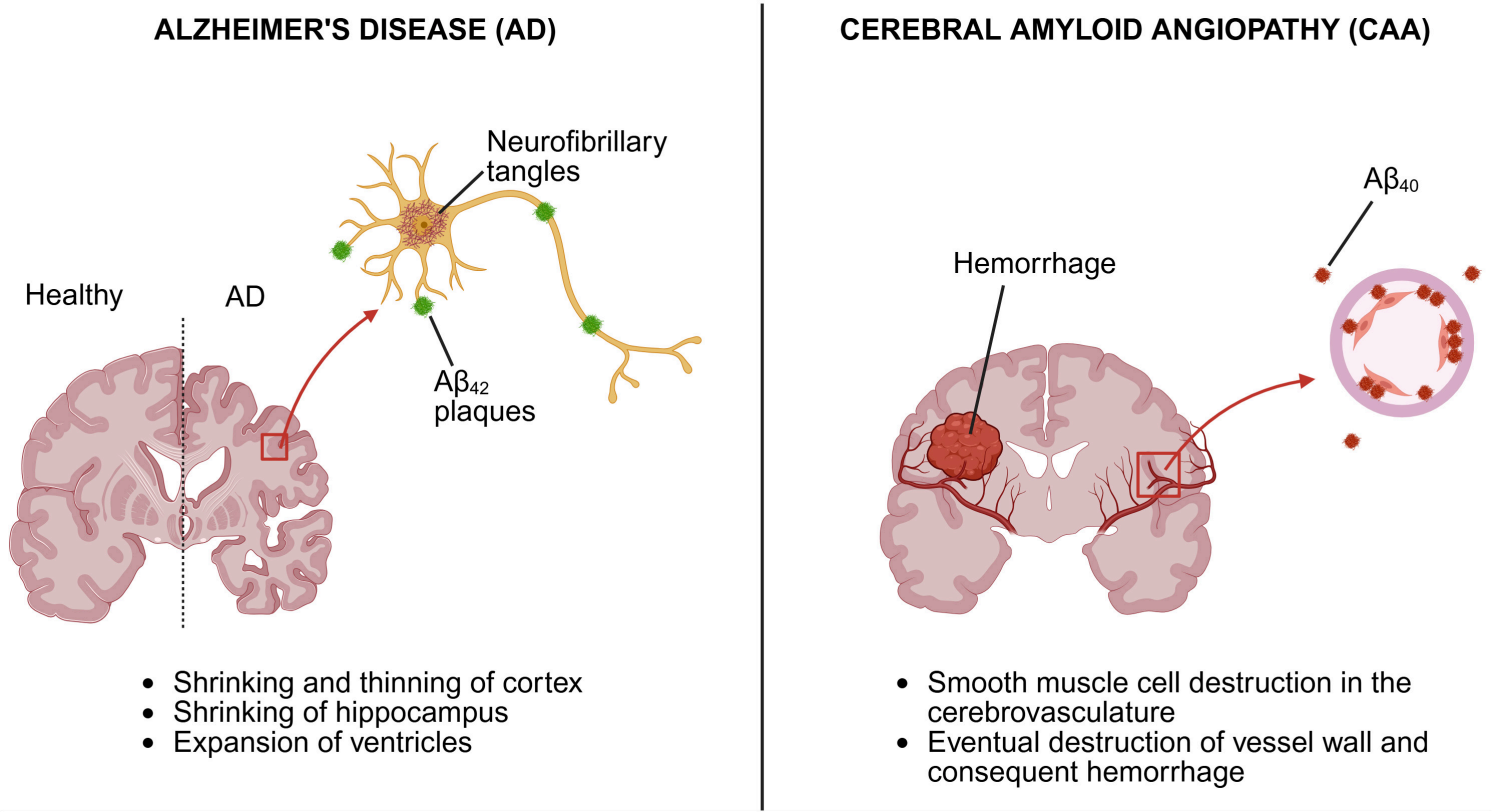
Alzheimer’s disease (AD) vs. Cerebral amyloid angiopathy (CAA) brain. AD and CAA are two independent conditions that often coexist, in which case CAA increases the severity of AD (Weller et al., 2008; Chwalisz, 2021). While AD affects mainly the neurons and is characterized by neurofibrillary tangles and Aβ plaques comprised mostly of Aβ_42_, CAA is restricted to the cerebral vasculature, in which Aβ accumulates in the tunica media layer of blood vessels leading to destruction of smooth muscle cells and the vessel wall as disease progresses (Weller et al., 2008; DeSimone et al., 2017; Greenberg et al., 2020). Created in BioRender. Speth, R. (2025) https://BioRender.com/a1ce2dt.

Cerebral amyloid angiopathy is strongly associated with AD (Figure 3; Saito et al., 2021). Both conditions involve the accumulation of Aβ peptides but differ in the site of accumulation. In AD, Aβ aggregates into parenchymal plaques primarily within the frontal and parietal association cortices, with later involvement of the hippocampus (Yoon and Jo, 2012; Greenberg et al., 2020). In contrast, CAA is marked by Aβ deposition within the vessel walls of small- to medium-sized arteries, arterioles, and capillaries of the leptomeninges and cerebral cortex (Weller et al., 1998, 2008; DeSimone et al., 2017; Chwalisz, 2021). In addition to differences in anatomical distribution, the predominant Aβ isoforms also differ. Aβ42 is the main component of parenchymal plaques in AD, whereas perivascular deposits in CAA are predominantly composed of Aβ40, although Aβ_42_ is also present (Gatti et al., 2020; Chwalisz, 2021).

**FIGURE 4:**
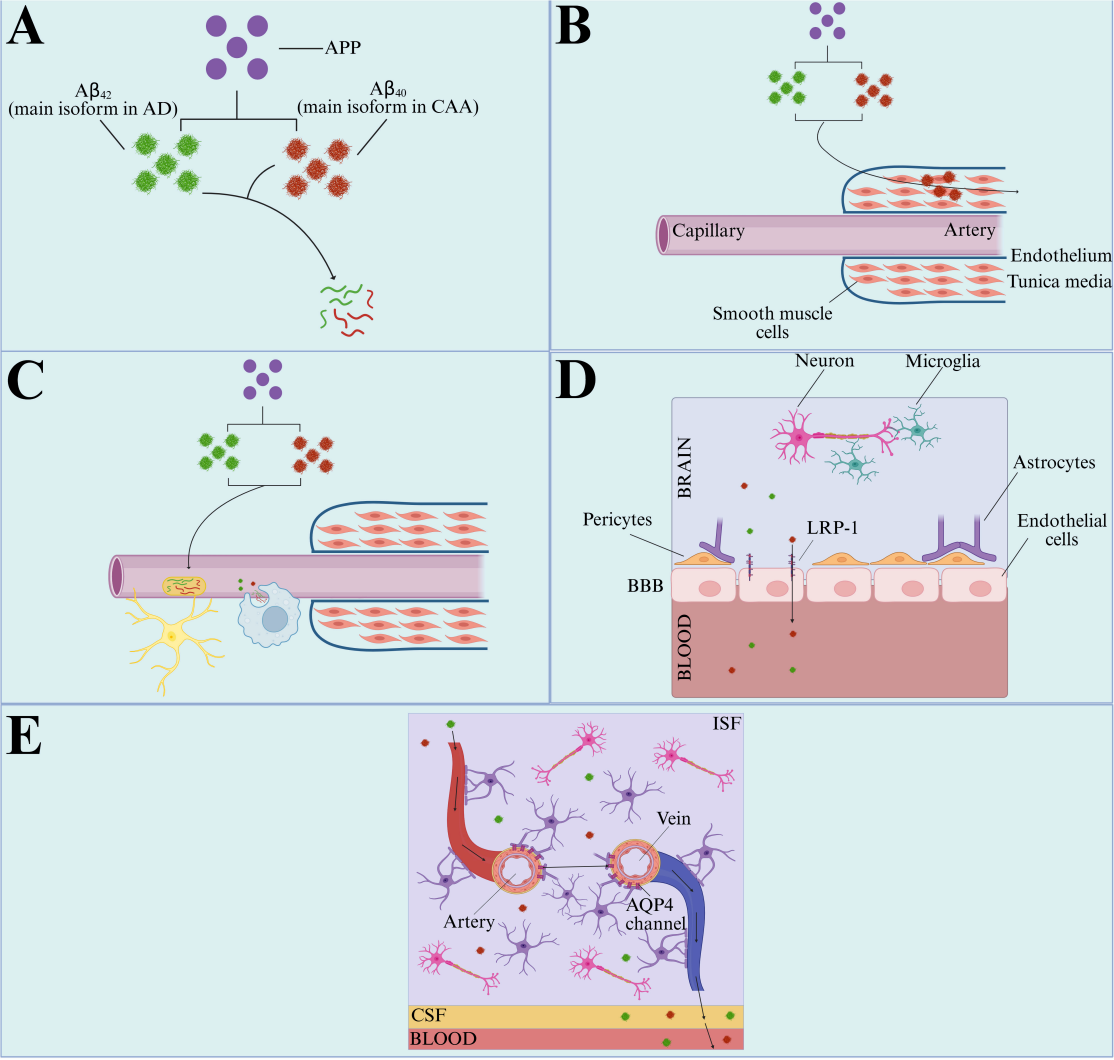
Amyloid-beta (Aβ) clearance pathways. Aβ peptides originate from the cleavage of APP by β- and γ-secretases (Selkoe et al., 1996; Wang et al., 2010). The length of these peptides varies with Aβ_40_ being predominant in CAA and Aβ_42_ in AD (Chwalisz, 2021). There are six main pathways of Aβ degradation: **(A)** degradation by metallopeptidase enzymes, insulin-degrading enzymes, and neprilysin, **(B)** perivascular clearance, when Aβ (particularly Aβ_40_) is taken up by the blood vessels and eliminated along with other soluble metabolites through the smooth muscle cells of the tunica media, **(C)** phagocytosis, when Aβ is taken up and degraded by astrocytes and macrophages, **(D)** blood-brain barrier (BBB) transport, when Aβ peptides are transported from the brain to the blood via LRP-1 receptors on the surface of endothelial cells that form the BBB, **(E)** glymphatic clearance, when clearance of Aβ peptides and other waste from the CSF to the blood is facilitated by astrocytic aquaporin-4 (AQP4) channels present in the astrocytic endfeet clefts attached to arteries and veins (Weller et al., 2008; Tarasoff-Conway et al., 2015; Vandal et al., 2015; DeSimone et al., 2017; Sousa et al., 2023). Created in BioRender. Speth, R. (2025) https://BioRender.com/a1ce2dt.

Although they share overlapping features, CAA and AD are distinct pathological entities; however, CAA pathology is present in approximately 90% of AD cases (Saito et al., 2021). In cases where they co-exist, CAA is believed to exacerbate both the pathological burden and clinical manifestations of AD. As vascular amyloid accumulates, the perivascular drainage route becomes blocked, reducing Aβ clearance and promoting its accumulation (Weller et al., 2008; Chwalisz, 2021). Routes of Aβ clearance are illustrated in Figure 4.

Recent research has highlighted the complex interplay between CAA, Aβ pathology, and tau-related neurodegeneration in AD, as well as their combined contributions to cognitive impairment. In Rabin et al. (2022) demonstrated that severe CAA pathology and high parenchymal Aβ levels were associated with increased tau deposition and accelerated cognitive decline. Godrich et al. (2024) attempted to replicate Rabin et al.’s (2022) findings using a larger dataset, confirming significant relationships with AD-related neuropathology and cognitive impairment. They reported that CAA interacts with neuritic plaques (senile plaques composed of both Aβ and immune cells) to influence neurofibrillary tangle burden and contributes to cognitive impairment by amplifying the effect of tangles on cognition, rather than acting independently (Godrich et al., 2024). More recently, Jury-Garfe et al. (2025) reported that in a CAA mouse model lacking endogenous tau (Tg-FDD/mTau^–/–^), the depletion of tau reduced amyloid deposition in the vasculature, prevented vascular damage, ameliorated motor and synaptic impairments, and decreased astrocytic reactivity, suggesting that downregulation of tau in CAA can function as a potential therapeutical strategy for treating patients with parenchymal and vascular amyloid deposition. Additionally, Kang et al. (2025) recently reported that lobar cerebral microbleeds (a CAA imaging marker) are associated with phosphorylated tau 217 (p-tau217), glial fibrillary acid protein (GFAP), and neurofilament light chain (NfL), which are AD plasma biomarkers that reflect tau hyperphosphorylation, astrocytic activation, and neuronal damage, respectively. Additionally, it was found that lobar cerebral microbleeds, in conjunction with p-tau217 and GFAP, were associated with cognitive impairment in a synergistic manner (Kang et al., 2025). A recent study of CAA patients with an AD profile present significant Cornu Ammonis (CA) 2-CA3 atrophy compared to CAA patients without an AD profile, suggesting that CA2-CA3 volume can serve as a radiological marker for identifying CAA-AD overlaps (Storti et al., 2025). This emerging evidence underscores the multifaceted relationships between CAA, Aβ pathology, and tau-driven neurodegeneration in AD, highlighting the need for an integrated approach to treatment.

In addition to exacerbating AD pathology and symptoms, CAA also complicates AD treatment with the newest anti-amyloid drugs (e.g., monoclonal antibodies). In treated patients, CAA increases the risk of amyloid-related imaging abnormality (ARIA), such as brain swelling (ARIA-E) and/or bleeding (ARIA-H), that can potentially exclude them from continued treatment (Sveikata et al., 2022; Agarwal et al., 2023; Sin et al., 2023). This is hypothesized to occur because these drugs break down parenchymal plaques and increase the mobilization of Aβ aggregates to the vasculature, increasing the severity of CAA (Hampel et al., 2023). A literature review by Barakos et al. (2022) reported that the incidence of ARIA-E has varied from 0.9% to 40.6%, while that of ARIA-H has varied from 0.5% to 28.4%. However, these ranges do not differentiate between different subgroups or drugs. Clinical trials have provided insight into the percentage of patients, within the general population of the studies, who developed ARIA after receiving specific anti-amyloid drugs (Table 3). Therefore, while anti-amyloid therapies show promise for AD treatment, their effectiveness is limited by CAA-related risks such as ARIA. This underscores the need for tailored treatment strategies that account for vascular amyloid burden.

**TABLE 3:** Amyloid-related imaging abnormality (ARIA) incidence during clinical trials of anti-amyloid drugs.

Drug	ARIA-E	ARIA-H
Lecanemab (van Dyck et al., 2023; Doran and Sawyer, 2024)	12.6%	17.3%
Aducanumab (Salloway et al., 2022; Doran and Sawyer, 2024)	35%	19%
Gantenerumab (Bateman et al., 2023; Salloway et al., 2025)	24.9%	22.9%
Donanemab (Zimmer et al., 2025)	19.8%–24.4%	27.2%–31.3%

### 3.5 Animal models of CAA

Given the high comorbidity of CAA pathology with AD (DeSimone et al., 2017; Chwalisz, 2021; Saito et al., 2021), and its contribution to conditions such as vascular cognitive impairment and stroke (Wattendorff et al., 1995), the development and use of animal models of CAA are critical for advancing our understanding of pathophysiological mechanisms and for evaluating the efficacy and safety of therapeutic interventions targeting CAA. Several mouse models that exhibit CAA exist (Table 4). In Davis et al. (2004) created a transgenic mouse model of CAA, the Tg-SwDI mouse. These mice exhibit the Dutch and Iowa mutations, as well as the Swedish K670N/M671L mutation, a double mutation that immediately precedes the aspartic acid at position 672 at the β-secretase cleavage site in the APP, causing Aβ_40_ and Aβ_42_ to be overproduced (Citron et al., 1994). Tg-SwDI mice show high levels of insoluble Aβ_40_ plaques (Robison et al., 2019), the main isoform present in CAA (Chwalisz, 2021), with modest levels of Aβ_42_. Aβ accumulation occurs primarily in the vasculature, though parenchymal accumulation is also observed, starting at ∼3 months of age with severity increasing over their lifespan (Xu et al., 2014; Paul et al., 2018; Rosas-Hernandez et al., 2020). Vascular amyloid burden is greatest in the subiculum, followed by the thalamus, but is almost absent in the cortex (Robison et al., 2019). Robust CAA-associated inflammation is exhibited in the Tg-SwDI mouse model (Fan et al., 2007a), which displays high expression of proinflammatory cytokines [e.g., tumor necrosis factor-alpha (TNF-α), interleukin-1beta (IL-1β), and interleukin-6 (IL-6)] (Robison et al., 2019). In behavior tests, these mice exhibit impaired spatial learning and memory in the Barnes maze test as early as 3 months of age (Xu et al., 2007; Robison et al., 2019, 2020).

**TABLE 4:** Mouse models that exhibit cerebral amyloid angiopathy (CAA) (Jäkel et al., 2017; Gatti et al., 2020; Vargas-George and Dave, 2022).

Mouse model • Availability • Primary use	Mutation*	CAA onset	Characteristics	Model limitations
Tg-SwDI • Commercial availability • Mechanistic inquiry and therapeutic testing	APP_770_ K670N/M671L, E693Q, D694N	3 months of age	• Microvascular Aβ deposition starting at 3 months of age; most abundant in the thalamus and subiculum • 50% of microvasculature affected at 12 months of age • 85%–90% of microvasculature affected at 24 months of age • Diffuse Aβ plaques formed in the cortex at 3 months of age • Minimal CAA on the frontotemporal cortex • No prominent hemorrhages • Impaired spatial learning and memory beginning at 3 months of age • Increased number of reactive astrocytes and active microglia at 3 months of age	• Tolerates larger amounts of toxic Aβ species than humans (Kokjohn and Roher, 2009) • Predominant CAA subtype (CAA Type 1) and some areas of CAA accumulation (e.g., thalamus) does not fully reflect that of humans (Miao et al., 2005) • Aβ plaques also observed; not a “pure” CAA model (Rodriguez-Lopez et al., 2025)
APPDutch • Commercial availability • Mechanistic inquiry and therapeutic testing	APP_751_ E693Q	22–25 months of age	• Irregular thickening of basement membrane • Hemorrhages at 29 months of age • Anxiety and learning deficits at 12 months of age	• Late onset (Jäkel et al., 2017)
APP/London (APP/Ld) • Commercial availability • Mechanistic inquiry and therapeutic testing	APP_695_ V717I	15–24 months of age	• Arteries affected more by CAA than veins • 70% of vascular amyloid load is in the leptomeningeal arterioles; 30% in cortical vessels • Presence of aneurysms at 20 to 24 months of age, without hemorrhages or altered cerebral blood flow • Early impairment in long-term memory at 3–4 months of age	• Primarily an AD model, although CAA is also present (Tanghe et al., 2010) • Absence of ICH due to CAA (Jäkel et al., 2017)
APP23 • Commercial availability • Mechanistic inquiry and therapeutic testing	APP_751_ K670N/M671L	9–12 months of age	• CAA predominantly affects arterioles and capillaries • Smooth muscle cells in the tunica media lost at 19 to 27 months of age • Microhemorrhages and decreased cerebral blood flow at 16 months of age • Deficits in spatial memory and passive avoidance at 3 months of age	• Although it mimics the progressive cognitive decline that is seen in humans, the earliest observed deficit does not reflect that of humans, requiring careful interpretation (Janssen et al., 2017) • Thalamus is one of the main sites of CAA development, which is not reflective of human CAA (Jäkel et al., 2017) • Majority of bleeding occurs in the cortex and thalamus, while in humans bleeding is predominately cortical (Jäkel et al., 2017)
ArcAβ • Academic availability • Mechanistic inquiry and therapeutic testing	APP_695_ E693G, K670N/M671L	9–15 months of age	• Dense-core plaque and intracellular Aβ deposits at 9–15 months of age • Smooth muscle cells of the tunica media disrupted at 16 to 22 months of age • Cerebral microbleed at 18 months of age • Astrogliosis at 6 months of age • Impaired blood-to-brain glucose transport at 9 to 13 months of age • Cognitive impairments starting at 4 months of age and being more robust at 6 months of age	• Mainly characterized by means of neuroimaging methods (Jäkel et al., 2017) • Several forms of Aβ found (e.g., Aβ_40_, Aβ_42_, Aβ_38_) (Jäkel et al., 2017)
PDAPP • Academic availability • Mechanistic inquiry and therapeutic testing	APP_770_ V717F	7–10 months of age	• Minimal CAA development • Behavioral deficits at 3 months of age • Object recognition impairment at 6–10 months of age	• Minimal CAA development compared to other models (Jäkel et al., 2017)
Tg2576 • Commercial availability • Mechanistic inquiry and therapeutic testing	APP_695_ K670N/M671L	7–10 months of age	• Microhemorrhages at 21–22 months of age • Larger vessels affected more severely by CAA than smaller vessels • Smooth muscle cells of the tunica media disrupted at 14 and 24 months of age • Vasomotor function reduced at 6 months of age • Altered blood flow • Plaque formation at 9 months of age • Learning and memory deficits at 6 months of age	• Less prominent CAA than other models (e.g., APP23) (Jäkel et al., 2017) • Better suited for AD research with associated CAA pathology (Jäkel et al., 2017)
TgCRND8 • Academic availability • Mechanistic inquiry and therapeutic testing	APP_695_ K670N/M671L, V717F	3–5 months of age	• Plaque formation at 3–6 months of age • Severe Aβ deposition in leptomeningeal vessels at 11 months of age • CAA affects microvessels and arterioles	• Extensive plaque development, suggesting that Aβ pathology is of mixed nature (Jäkel et al., 2017)
APP/PS1 • Commercial availability • Mechanistic inquiry and therapeutic testing	APP_swe_/PSEN_1de9_	6 months of age	• Extensive plaque formation at 6–7 months of age • Higher level of Aβ_42_ • Learning and memory deficit at 17 weeks of age	• Less prominent CAA than other models (e.g., Tg2576) (Jäkel et al., 2017) • Low vasculotropic levels of Aβ_40_ (Jäkel et al., 2017)
E22ΔAβ • Academic availability • Mechanistic inquiry and therapeutic testing	APP_695_ K670N/M671L, E693_del_	24 months of age	• Absence of parenchymal plaques • Vascular Aβ deposits at 24 months of age • Cognitive impairment at 3–6 months of age	• Late onset (Jäkel et al., 2017)
Tg-ArcSwe • Academic availability • Mechanistic inquiry and therapeutic testing	APP K670N/M671L, E693G	8 months of age	• Development of plaques at 5–6 months of age and CAA at 8 months of age • CAA detected at 8 months of age and abundant at 15 months of age in capillaries, arterioles, and arteries	• Absence of ICH (Skaaraas et al., 2021)
APP knock-in • Commercial availability • Mechanistic inquiry and therapeutic testing	APP K670N/M671L, I716F	18 months of age	• Anxiety-like behavior • Learning and memory deficits at 6 months of age	• Aβ plaques also observed; not a “pure” CAA model (Xia et al., 2022)
eNOS^+/−^ • Academic availability • Mechanistic inquiry and therapeutic testing	eNOS^+/–^	6 months of age	• Microhemorrhages at 18 months of age • Cognitive deficits at 18 months of age	• Late onset of cognitive deficits (Vargas-George and Dave, 2022)

*APP subscripts correspond to the APP isoform in which mutations are observed.

Van Nostrand et al. (2001) have more recently created rat models carrying the familial CAA mutations, including the rTg-DI (Davis et al., 2018, 2022; Popescu et al., 2020; Zhu et al., 2020; Lee et al., 2021; Schrader et al., 2021, 2022a; 2022b,2024a; 2024b; Stanisavljevic et al., 2022; Vervuurt et al., 2022, 2024; Koundal et al., 2024) and rTg-D (Irizarry et al., 2025) models, exhibiting Type I and Type II CAA, respectively. In addition to these, other rat models exist (e.g., F344-AD, and TgAPP21), as do naturally occurring models [e.g., primates (baboon, cynomolgus monkeys, gray mouse lemur, rhesus monkeys, squirrel monkeys, and velvet monkeys), cats, and dogs] (Jäkel et al., 2017).

### 3.6 Nomenclature and localization

It is important to note that this review focuses on CAA caused by the accumulation of Aβ in cerebral vasculature, as the term “amyloid” is generic with respect to the identity of the proteins or peptides that make up amyloid aggregates. Specifically, they have “characteristic fibrillar electron microscopic appearance, typical X-ray diffraction pattern and histological staining reactions, particularly affinity for the dye Congo red with resulting green birefringence” (Westermark et al., 2007). According to the 2024 Nomenclature meeting of the International Symposium for Amyloidosis (Buxbaum et al., 2024), the terms APP and plaques, used to describe the amyloid aggregates that are associated with AD, are technically incorrect. This most recent Symposium identified 42 different proteins that are associated with amyloidosis that all meet the criterion of “APP.” Thus, the recommendation of the Symposium is that the APP from which Aβ is derived should be called Aβ precursor protein (AβPP). Additionally, the Symposium recommended avoiding the word plaque in AD, as it refers to a flat object, whereas the amyloid aggregates associated with AD are globoid in shape. However, the Symposium recognized that the near universal terminology of APP and Aβ plaques in the AD literature is unlikely to be changed due to technical inaccuracy.

While Aβ deposits have been found in non-neural tissues surrounding arterioles and microvessels, they were reported to be extracellular (Joachim et al., 1989) precluding a determination of amyloid angiopathy. Moreover, non-neural Aβ was deemed unlikely to cause clinical dysfunction akin to that of CAA.

A variant CAA arises from the formation of aggregates of transthyretin (TTR), a protein derived from the liver and choroid plexus (Brunet de Courssou et al., 2025). However, this variant of CAA is far rarer than that resulting from the accumulation of Aβ aggregates. It is associated with a naturally occurring mutation in the transthyretin gene that has a neuropathological phenotype distinct from that of CAA involving Aβ aggregates (Sekijima et al., 2016). Hereditary TTR amyloidosis affects peripheral and autonomic nervous system and heart, although effects in the kidneys, eyes, and digestive system can also be observed (Brunet de Courssou et al., 2025). In cases where TTR amyloidosis results in CAA, patients tend to present more focal central neurological symptoms, seizures, and memory complaints, compared to patients with TTR amyloidosis but no CAA (Brunet de Courssou et al., 2025).

### 3.7 Therapeutic approaches

Currently, there are no FDA-approved medications that specifically treat or prevent CAA (Kozberg et al., 2021). Management of the disease mainly revolves around reducing the risk of first-time or recurrent lobar ICH and controlling symptoms (Gatti et al., 2020; Kozberg et al., 2021). However, ongoing research is exploring strategies to lower Aβ deposition during different disease stages, such as enhancing perivascular clearance of Aβ through pharmacological and non-pharmacological interventions (Kozberg et al., 2021; Cozza et al., 2023).

The bioactive flavanonol taxifolin, also known as dihydroquercetin, has antioxidant and anti-inflammatory properties. It has been reported to disaggregate Aβ fibrils and inhibit their formation, making it a potentially safe therapeutic agent to treat CAA (Sato et al., 2013a; Saito et al., 2021). The properties of taxifolin have been demonstrated by transmission electron microscopy imaging in a mouse model of CAA (Sato et al., 2013a,b; Saito et al., 2017), and by thioflavin T fluorescence assays showing disintegration of Aβ fibrils with inhibition of new ones. This could facilitate Aβ clearance and restore cerebrovascular reactivity and spatial memory (Sato et al., 2013a; Saito et al., 2017). In wild-type mice injected with oligomeric Aβ into the hippocampus, taxifolin prevented spatial memory deficits that are typically induced by the oligomer (Wang et al., 2018). Although taxifolin has shown promising results in mouse models, its effects in humans with CAA have yet to be evaluated in clinical trials.

Cerebrolysin^®^, a parenterally administered porcine brain-derived neurotrophic peptidergic drug (Plosker and Gauthier, 2009) indicated for the treatment of central nervous system (CNS) disorders in Europe and Asia^2^, has shown positive effects in the treatment of AD in both animal models and humans. However, Cerebrolysin^®^ does not have FDA approval in the United States. Treatment with Cerebrolysin^®^ was shown to decrease Aβ deposition around the blood vessels, reduce perivascular microgliosis and astrogliosis, increase the expression of vascular fitness markers, reduce changes in the endothelial and smooth muscle cells, and preserve basal membranes and intracellular junctions in mThy1-hAPP751 Tg mice (a mouse model of AD) treated with Cerebrolysin^®^ for 3 months, starting at 7 or 12 months of age (Rockenstein et al., 2005). Additionally, Cerebrolysin^®^ significantly reduced amyloid burden and ameliorated synaptic impairments in the frontal cortex of 5-months-old mThy1-hAPP751 Tg mice (Rockenstein et al., 2002). When this strain of mice was treated with Cerebrolysin^®^ for 3 months, starting at 3 or 6 months of age, results showed improvement in behavioral performance, synaptic regeneration, and reduction in the proportion of neurons displaying DNA fragmentation (Rockenstein et al., 2003). In humans with advanced AD, it has been shown that treatment with Cerebrolysin^®^ in combination with donepezil (an acetylcholinesterase inhibitor prescribed to mitigate AD symptomatology) normalizes the levels of serum vascular endothelial growth factor (VEGF) – which increases with AD severity, playing a paradoxical role in the disease (Ali and Bracko, 2022) – while significantly improving cognitive function; however, no therapeutic effects were observed in mild to moderate AD cases (Alvarez et al., 2020).

Tramiprosate (Cerebril^®^), an orally administered low molecular weight aminosulfonate compound, binds to soluble Aβ_42_ at amino acids Lys16, Lys28, and Asp23, stabilizing it and reducing plaque formation and providing neuroprotection (Greenberg et al., 2006; Manzano et al., 2020; Cozza et al., 2023). In TgCRND8 mice treated with subcutaneous injections of 30 or 100 mg/kg/day Tramiprosate for 8–9 weeks, the drug was found to cross the BBB; reduce the % area occupied by plaques by 29% in both groups; reduce the number of plaques by 24% and 18%, respectively; reduce Aβ_40_ plasma levels by 37% and 61%, respectively; and reduce Aβ_42_ plasma levels by 31% and 67%, respectively (Gervais et al., 2007). Additionally, fifth generation progeny obtained from initially crossing TgCRND8 mice with C57BL/6 mice, and then alternately crossing the progeny with FVB or C57BL/6 mice up to the fifth generation, were treated for 9 weeks with 500 mg/kg/day Tramiprosate (Gervais et al., 2007). Results showed that brain levels of soluble Aβ_40_, insoluble Aβ_40_, soluble Aβ_42_, and insoluble Aβ_40_ were reduced by 30%, 31%, 25%, and 22%, respectively (Gervais et al., 2007). In phase 1 clinical trials, Tramiprosate was administered for up to 1 week with favorable pharmacokinetic properties and no major safety issues. However, minor side-effects, particularly nausea and vomiting, were shown to be dose-dependent (Greenberg et al., 2006). In phase 2 clinical trials, Tramiprosate showed no significantly beneficial effects on cerebral microbleeds, but data suggested that the drug could be safely given to CAA patients due to no major safety issues being reported (Greenberg et al., 2006; Aisen et al., 2011; Saito et al., 2021). In a study by Gauthier et al. (2009), 508 patients underwent volumetric MRI scanning to assess disease modification of the hippocampus while receiving 100 or 150 mg Tramiprosate twice daily for 78 weeks. Results showed that treatment, particularly with the 150 mg dose, slowed down hippocampal atrophy and revealed some evidence of positive effects on cognition, based on the Alzheimer’s Disease Assessment Scale-cognitive subscale (ADAS-cog) (Gauthier et al., 2009).

In cases of CAA-ri, patients respond well to immunosuppressive therapy, typically corticosteroids, although cyclophosphamide, azathioprine, mycophenolate mofetil, methotrexate, and immunoglobulin are also commonly used (Auriel et al., 2016; Meng et al., 2024). According to Kirshner and Bradshaw (2015), improvement is observed within a few weeks of treatment and occurs in approximately 72% of patients treated with immunosuppressants. Although the exact duration of treatment is yet to be determined and varies depending on the patient, a typical course of treatment consists of a high-dose steroid pulse given intravenously followed by a prolonged steroid taper of at least 6 months (Kirshner and Bradshaw, 2015; Kozberg et al., 2021). Despite the efficacy of the treatment, cases of recurrence have been reported. A retrospective cohort study by Regenhardt et al. (2020) following 48 people suffering from CAA-ri found that recurrence occurred in 26% of those who received any immunosuppressive agent, compared to 71% of those who did not receive any. Recurrence can occur at the initial inflammation site and/or additional sites (DiFrancesco et al., 2015; Kozberg et al., 2021).

A novel investigational approach to mitigate CAA and AD disease progression is the use of an RNA interference (RNA_*i*_) therapeutic called mivelsiran (ALN-APP). The drug, which is administered intrathecally, is designed to lower Aβ levels by decreasing APP synthesis (Cohen et al., 2024). According to the manufacturer (Alnylam^®^ Pharmaceuticals), the drug acts by decreasing APP messenger RNA (mRNA) in the central nervous system, which reduces both APP synthesis and APP-derived cleavage products, such as Aβ^3^. During phase 1 clinical trials (NCT05231785), the safety, tolerability, pharmacokinetics, and pharmacodynamics of mivelsiran in patients with early-onset AD was evaluated, after single doses of 25, 50, 75 mg, or placebo were administered for 6 months. Interim results showed that 50 and 75 mg of mivelsiran are well-tolerated and produce sustainable reductions in the levels of soluble APP in the CSF, as well as Aβ_42_ and Aβ_40_ (Cohen et al., 2024). Phase 2 clinical trials (cAPPricorn-1/NCT06393712) will evaluate the safety, tolerability, and pharmacodynamics of mivelsiran in adult patients with sCAA and Dutch-type CAA, as well as evaluate its effects on markers of CAA progression, such as rate of new lobar cerebral microbleeds. If approved, mivelsiran will offer a new mechanism of action for drugs used in the treatment of CAA and AD, as it will focus on reducing APP synthesis and, as a consequence, Aβ isoforms, instead of focusing on Aβ peptide/plaques clearance after they have already formed, like currently approved drugs do.

It is important to note that there are certain medications that should be avoided following CAA diagnosis. The use of anticoagulant and antithrombotic agents has been generally discouraged, as these significantly increase the risk of hemorrhagic stroke in those with CAA (Banerjee et al., 2017; Sheth, 2022; Merella et al., 2023). Similarly, antiplatelet agents have been associated with an increased number of cerebral microbleeds and a higher risk of ICH recurrence in patients with CAA (Vernooij et al., 2009; Biffi et al., 2010). These agents should be avoided unless there is a compelling clinical indication for their use, such as a high risk of ischemic stroke due to blood clot formation that clearly outweighs the risk of ICH (Kozberg et al., 2021; Merella et al., 2023).

## 4 Lifestyle and CAA

### 4.1 Diet and metabolic disorders

Emerging evidence suggests that dietary factors may influence the development and progression of CAA, potentially through effects on vascular health, inflammation and oxidative stress, and amyloid metabolism. In recent years, ketogenic diets (high-fat, low carbohydrate, moderate protein intake) have gained attention for their ability to promote brain health, improve cognition, and protect against other cerebrovascular and neurodegenerative diseases (Pulido-Correa et al., 2025). Pulido-Correa et al. (2025) have recently shown that consumption of a ketogenic diet increased exploratory activity and improved spatial learning and memory in male Tg-SwDI mice. These cognitive-behavioral benefits were associated with an attenuation of parenchymal amyloid pathology and increased hippocampal neurogenesis; however, cerebrovascular amyloid levels were unchanged (Pulido-Correa et al., 2025).

The Mediterranean diet has received considerable attention in recent years due to its potential neuroprotective and cardiovascular benefits (Guasch-Ferré and Willett, 2021; Laffond et al., 2023; Pant et al., 2023; Picone et al., 2024; Fekete et al., 2025). This diet is characterized by a high intake of fruits, vegetables, whole grains, legumes, nuts, and olive oil; moderate consumption of fish and poultry; low intake of red meat and processed foods; and limited consumption of dairy products and sweets. The MIND (Mediterranean-DASH Diet Intervention for Neurodegenerative Delay) diet, a hybrid of the Mediterranean and DASH diets, was specifically developed to reduce the risk of AD and cognitive decline. The MIND diet is similar to the Mediterranean diet but puts a stronger emphasis on specific brain-healthy foods like leafy greens and berries. Both diets have been consistently associated with a reduced risk of cognitive decline and dementia across numerous epidemiological and clinical studies (van den Brink et al., 2019); however, little is known about their potential for protecting against CAA, specifically. A study in Tg-SwDI mice explored the potential benefits of a diet rich in extra-virgin olive oil, a major component of the Mediterranean and MIND diets. Consumption of this diet improved cognitive-behavioral function and attenuated both vascular and parenchymal Aβ deposition, with evidence for reduced Aβ production and enhanced clearance pathways (Qosa et al., 2015). Similarly, it was found that a diet enriched with docosahexaenoic acid [an omega-3 fatty acid obtained from food (e.g., fatty fish) and fish oil supplements, that is essential for brain growth and development (Horrocks and Yeo, 1999)] reduces vascular Aβ accumulation, microhemorrhages, and inflammation in Tg2576 mice (Hur et al., 2018). A proposed clinical trial (NCT06933212) will be investigating the ability of Mediterranean diet to reduce cognitive decline in patients with CAA.

Several studies have suggested that consumption of a high-fat diet, rich in saturated and/or hydrogenated fats, as well as in simple and/or refined carbohydrates, can contribute to cognitive decline and the development and progression of AD (Kalmijn et al., 1997, 2004; Morris et al., 2003, 2006; Eskelinen et al., 2008; Kanoski and Davidson, 2011; Francis and Stevenson, 2013; Cordner and Tamashiro, 2015); however, less is known about its effects on CAA and related outcomes. In a study by Lin et al. (2016), 10 weeks of high fat diet increased cerebrovascular Aβ accumulation, elevated oxidative stress in the hippocampus, and exacerbated cognitive deficits in 5xFAD mice. Notably, high-fat diet selectively increased Aβ deposition in the microvessels of the hippocampus and in cortical arteries without affecting parenchymal Aβ levels (Lin et al., 2016). These pathological changes occurred despite minimal weight gain and only mild glucose intolerance in the high fat diet fed group of 5xFAD mice (Lin et al., 2016). Small sample sizes may have impacted the ability for these metabolic effects to reach significance; however, it was also noted that 5xFAD mice exhibited a blunted metabolic response to high fat diet compared to wild-type controls (Lin et al., 2016). It has also been reported that high fat diet-induced obesity did not increase levels of soluble or insoluble Aβ40 or Aβ42 in Tg-SwDI mice; however, this study did not distinguish between parenchymal and vascular Aβ accumulation (Zhang et al., 2013). High fat diet-fed Tg-SwDI mice also exhibited a localized increase in reactive microglia in the CA1 region of the hippocampus, as well as an increase in IL-6 expression in the striatum (Zhang et al., 2013).

Along with other environmental and genetic risk factors, consumption of a high fat diet can contribute to an increased risk for metabolic disorders, including prediabetes and type 2 diabetes mellitus (T2DM), dyslipidemia, and hypertension. Studies have suggested that metabolic disorders contribute to AD and CAA pathology. Vargas-Soria et al. (2022) reported that high fat diet-induced prediabetes accelerated amyloid accumulation in small to medium- sized vessels and increased Aβ plaque load in APPswe/PS1dE9 mice. Additionally, this study crossed APPswe/PS1dE9 with db/db mice (T2D model), resulting in T2D; findings suggest that T2DM enhanced Aβ accumulation in medium to larger- sized vessels, favoring vascular over parenchymal accumulation (Vargas-Soria et al., 2022). Additionally, both prediabetes and T2D were associated with attenuated vessel diameter, oxidative stress, and MMP activation (Vargas-Soria et al., 2022).

Several observational studies have shown an association between high cholesterol in late life and increased risk for mild cognitive impairment, AD, and vascular dementia (Kivipelto et al., 2001; Yaffe et al., 2002; Whitmer et al., 2005; Solomon et al., 2007, 2009). In humans, high cholesterol is associated with greater vascular Aβ (CAA), as well as an increase in neuritic plaques, diffuse plaques, and neurofibrillary tangles (Hawkes et al., 2015; Xu et al., 2020). However, in mice, this relationship has not yet been observed. Hohsfield et al. (2014) showed that hypercholesterolemia induced by prolonged consumption of a 5% cholesterol diet did not induce vascular amyloid accumulation in wild-type (C57BL/6J) nor 3xTg-AD mice, though intraneuronal Aβ and neuroinflammation were increased in the latter. However, measurements could not be conducted at an older age, as 3xTg-AD mice on the cholesterol diet did not survive up to 20 months (Hohsfield et al., 2014), making it impossible to know if prolonged 5% cholesterol diet would have effects similar to those seen in humans later in life.

Atherosclerosis is a chronic, progressive vascular disease characterized by the accumulation of lipids, inflammatory cells, and extracellular matrix components in medium and large arteries. This results in the formation of atherosclerotic plaques, which can lead to arterial wall thickening, loss of elasticity, and luminal narrowing. Over time, these plaques can calcify, rupture, or induce thrombosis, contributing to the development of cardiovascular events. Atherosclerosis may also contribute to AD and CAA pathology. Wang et al. (2021) showed that in 3xTg-AD mice (AD model) fed a high-fat diet, atherosclerosis induces the formation of platelet-associated fibrillar Aβ aggregates; increases CAA burden, tau pathology, and neuronal loss; impairs cerebral blood flow, and exacerbates cognitive deficits.

### 4.2 Blood pressure (BP)

As defined by the World Health Organization, hypertension (also known as high blood pressure) occurs when the pressure in the blood vessels is ≥140/90 mmHg. It can be asymptomatic and is directly related to older age, genetics, overweight, sedentarism, high-salt diet, and high alcohol consumption.^4^ Hypertension can impact vascular clearance of Aβ and increase inflammation and the risk of ICH (Rius-Pérez et al., 2018; Singh et al., 2022). Studies have shown that BP management can provide protection against CAA-related ICH (Arima et al., 2010; Biffi et al., 2015), and that patients with CAA should ideally maintain their BP lower than 120/80 mmHg (Li et al., 2017; Kozberg et al., 2021). However, a study by Sin et al. (2024) analyzing data from 2,510 participants in the National Alzheimer’s Coordinating Center who had systolic BP measurements recorded before a CAA-related death, found no evidence of systolic BP being associated with CAA. Additionally, a study by Zhang et al. (2020) reported that hypertension may not be associated with worse outcomes in patients who suffered a CAA-associated ICH. In fact, hypertensive patients exhibited a 50% lower risk of death 3 months after a CAA-associated ICH compared to normotensive patients (Zhang et al., 2020). An alternative explanation provided by the authors is that prolonged use of antihypertensive medications in patients with hypertension may provide protection against CAA and its complications (Zhang et al., 2020), as was discussed previously.

Limited rodent studies have been performed investigating the role of hypertension in the progression of CAA. In 10-months-old rTg-DI rats (a rat model of CAA type I), non-pharmacological hypertension was shown to cause cognitive-behavioral deficits and the redistribution of vascular Aβ, reducing microvascular accumulation in the hippocampus and thalamus and increasing accumulation in the surface pial vessels (Stanisavljevic et al., 2022). Additionally, while hypertension increased the occurrence of vessel occlusions in the thalamus of the rTg-DI rats, it did not alter the number of cerebral microbleeds nor the extent of gliosis in the thalamus (Stanisavljevic et al., 2022). Due to conflicting results and few studies in rodent models, the relationship between BP and CAA requires further study.

### 4.3 Physical activity and cognitive stimulation

Studies have established that exercise, as well as cognitively stimulating activities, can reduce the risk of dementia in humans (Cotman and Berchtold, 2002; Pope et al., 2003; Lange-Asschenfeldt and Kojda, 2008; Radak et al., 2010), healthy mice (Kempermann et al., 2002; Benaroya-Milshtein et al., 2004; Ekstrand et al., 2008; Birch et al., 2013), and other rodent models of AD (Arendash et al., 2004; Jankowsky et al., 2005; Lazarov et al., 2005; Costa et al., 2007; Hu et al., 2010; Andrade-Guerrero et al., 2023). In humans, it has been shown that exercise provides protective effects that are pathology-specific in the structure and function of aging brains (Memel et al., 2021). For example, exercise provided resilience against arteriosclerosis, atherosclerosis, and Aβ; while providing resistance against cerebrovascular dysfunction and Lewy body disease (Memel et al., 2021). Additionally, studies have shown that microinfarcts, neuronal loss, and white matter pathologies are associated with lower levels of total daily physical activity in older adults (Buchman et al., 2018).

Numerous studies have reported that exercise is neuroprotective in mouse models of AD. For example, a study by Andrade-Guerrero et al. (2023) using 13-months-old 3xTg-AD mice also showed that voluntary physical exercise reduced Aβ pathology and improved cognitive function. In a study by Maliszewska-Cyna et al. (2020), it was seen that sedentary TgCRND8 (another mouse model of AD) present lower capillary density in the cortex and higher capillary density in the hippocampus compared to non-transgenic controls. In AD brains, increased angiogenesis (formation of new blood vessels) has been observed in the hippocampus, midfrontal cortex, substantia nigra pars compacta, and locus coeruleus (Jefferies et al., 2017). Due to impaired cerebral blood flow, the state of hypoxia leads to compensatory angiogenesis through the upregulation of pro-angiogenic factors (e.g., VEGF). However, in AD brains, downstream molecules to VEGF destabilize the vessel wall of mature vessels and new vessels are not able to mature, leading to the formation of a leaky blood vessel network, which results in vascular remodeling and structural changes that compromise the integrity of the blood-brain barrier (BBB) (Jefferies et al., 2017). In the previously mentioned study by Maliszewska-Cyna et al. (2020), when the mice were exposed to 3 months of voluntary running, microvascular morphology in the affected regions were normalized and short-term spatial memory was significantly improved, despite no effects on Aβ pathology being observed. Also, in TgCRND8 mice, it was shown that the benefits of exercise extend to their offspring (Herring et al., 2012). Voluntary running during pregnancy significantly reduced Aβ plaque burden and amyloidogenic processing of APP, increased angiogenesis, improved neurovascular function, and reduced microgliosis, inflammation, and oxidative stress in transgenic offspring (Herring et al., 2012).

To date, few human studies have examined the relationship between exercise and CAA. A post-mortem analysis of 428 participants from the RUSH Memory and Aging Project reported no relationship between physical activity levels and CAA burden at autopsy (Buchman et al., 2018). However, physical activity was assessed over a relatively short duration (up to 10 days) and late in life, with 90% of this data collected within 36 months before death, potentially limiting the ability to detect long-term associations (Buchman et al., 2018). Additionally, it was found that the presence of macroinfarcts, nigral neuronal loss, and white matter pathologies are associated with lower levels of daily physical activity (Buchman et al., 2018). Of note, in the population studied, 72% were females, 23% presented the ApoE ε4 genotype, 39% had clinical dementia, and 1.06% had CAA diagnosed postmortem (Buchman et al., 2018).

In line with these findings, Robison et al. (2019) found that long-term access to a running wheel for 1, 3, or 12 h per day from 4 to 12 months of age failed to reduce CAA in mixed sex Tg-SwDI mice. However, anxiety-like behavior, increased sociability and short-term spatial memory, and decreased expression of pro-inflammatory cytokines, were observed following this exercise intervention (Robison et al., 2019). In contrast, a case study described a 55- years-old woman with CAA who experienced a temporal intracerebral hemorrhage likely during treadmill exercise (Malik et al., 2017), raising concerns that physical exertion might exacerbate cerebrovascular vulnerability in individuals with CAA. However, no detail about the level of CAA severity was provided. Together, these findings highlight the current uncertainty regarding the effects of exercise on CAA, underscoring the need for longitudinal studies to clarify whether exercise confers risk or benefit in this context.

Cognitive training has been widely studied for its potential to delay or mitigate cognitive decline and dementia, particularly in older adults. Some observational studies report that individuals who engage in lifelong cognitively stimulating activities have a lower risk of developing AD or other dementias (Schultz et al., 2015; Wu et al., 2023). Cognitive training involves structured tasks designed to improve specific cognitive domains such as memory, attention, executive function, and processing speed. Several randomized controlled trials (RCTs) (e.g., the ACTIVE trial) and meta-analyses have demonstrated that cognitive training can produce modest improvements in the trained domains that can be sustained for months to years post-training in older adults (Tennstedt and Unverzagt, 2013; Basak et al., 2020). However, RCTs in populations at risk for dementia (e.g., patients with mild cognitive impairment) have shown mixed results (Maffei et al., 2017; Orgeta et al., 2020). Cognitive training is hypothesized to protect against dementia by enhancing cognitive reserve and promoting neuroplasticity and increasing neural efficiency in trained brain regions (Park and Bischof, 2013).

Cognitive training has also been tested in animal models (Billings et al., 2007; Martinez-Coria et al., 2015; Anderson et al., 2017; Mehla et al., 2023). A study conducted in APP knock-in mice found that cognitive training at 3, 6, and 9 months of age improved cognitive and cholinergic functions and reduced Aβ load and microgliosis (Mehla et al., 2023). Similarly, a comparative study done in 3xTg-AD mice, spatially trained and tested every 3 months from 2 to 18 months of age, found training delays the decline in spatial memory and the development of AD pathology (Billings et al., 2007). However, a study by Anderson et al. (2017) found that Tg-SwDI and 5xFAD mice submitted to a high-intensity 4-months cognitive training presented little evidence of reduced Aβ pathology (including CAA) or cognitive improvement.

There are limited studies on the effects of combined interventions. A study by Robison et al. (2020) examined the effects of environmental enrichment (social and cognitive enrichment, as well as access to a running wheel for exercise) and its individual components from 4 to 8 months of age in female Tg-SwDI mice. All enrichment conditions resulted in an attenuation of vascular amyloid accumulation in the thalamus; however, cognitive-behavioral benefits varied by group, with the fully enriched environment exerting the widest range of benefits. These findings suggest that cognitive, social, and physical enrichment, particularly when combined, can be beneficial for targeting CAA and reducing the risk of cognitive decline (Robison et al., 2020).

Results from the landmark FINGER randomized controlled trial also support a multimodal interventional approach, combining cognitive training with exercise, dietary modification, social engagement, and management of vascular risk factors in at-risk older adults (Ngandu et al., 2015). However, this trial did not assess CAA specifically. In July 2025, a press release from the Alzheimer’s Association International Conference, provided updates on the U.S. Study to Protect Brain Health through Lifestyle Intervention to Reduce Risk (U.S. POINTER), a phase 3, five-site, 2-years, single-blinded randomized clinical trial of two lifestyle interventions in older adults at risk for dementia. U.S. POINTER assessed whether the results observed in the FINGER trial can be generalized to a larger, more diverse, population in the U.S. that are at risk for developing dementia and cognitive decline^5^. Participants were divided into self-guided and structured lifestyle intervention groups, which focused on increasing exercise, improving nutrition, cognitive and social challenge, and monitoring health^6^. According to the press release, participants in both groups displayed improved cognition, although those in the structured intervention group had greater improvements. Results were consistent across age, sex, ethnicity, heart health status, and ApoE ε4 genotype^7^.

### 4.4 Stress

Studies have reported that chronic stress and stress-related disorders increase the risk of cognitive decline and dementia (Peavy et al., 2012; Kim et al., 2023; Wallensten et al., 2023). Several studies in AD rodent models have demonstrated that stress exacerbates AD-related pathology and cognitive deficits (Carroll et al., 2011; Baglietto-Vargas et al., 2015; Stuart et al., 2017; Peterman et al., 2020; Shlomi-Loubaton et al., 2025); however, less direct evidence exists for its effects on CAA specifically. A study conducted by Huang et al. (2024) showed that chronic stress exacerbated CAA in Tg-SwDI mice, along with BBB injury and myelin loss. Chronic stress promoted vascular Aβ accumulation through neutrophil activation. Stress-induced norepinephrine enhanced STAT6 signaling, triggering NET formation and NETosis, contributing to vascular amyloid deposition. Inhibiting neutrophil recruitment or suppressing NETs formation reduced CAA severity (Huang et al., 2024). Another study using APPV717I-CT100 mice overexpressing human APP-CT100 containing the London mutation (V717I) found that chronic mobilization stress also increased vascular amyloid accumulation, accompanied by an acceleration of cognitive impairment (Jeong et al., 2006).

### 4.5 Sleep

Sleep disorders and disturbances have been linked to poor brain health and more rapid cognitive decline (Atayde et al., 2020; Jackson et al., 2020). Sleep deprivation negatively affects attention, executive function, and short-term memory, and causes impairments in the glymphatic system, which facilitates waste removal (e.g., Aβ) that typically has enhanced activity during sleep (Durmer and Dinges, 2005; Gottesman et al., 2024).

Human and animal studies have further explored the connection between sleep disturbances and brain health. A brain autopsy of 315 patients from the Rush Memory and Aging Project showed that greater sleep fragmentation was associated with increased severity in arteriosclerosis and higher number of subcortical infarcts (Lim et al., 2016). In APP/PS1 mice with induced chronic sleep deprivation, it was shown that sleep deprivation exacerbated vascular lesions, with extensive Aβ deposits being observed, and increased expression of the lysine specific demethylase 6B (KDM6B) histone, which is associated with neuronal injury and inflammation (Yu et al., 2025). Furthermore, knockdown of the KDM6B histone ameliorated sleep deprivation-induced memory impairment, neuronal injury, and vascular lesions, and inhibited neuronal cytotoxicity of Aβ_42_ (Yu et al., 2025).

### 4.6 Smoking

Smoking is considered a modifiable risk factor for AD (Barnes and Yaffe, 2011; Daviglus et al., 2011; Etgen et al., 2011; Durazzo et al., 2014). It is strongly associated with oxidative stress in the brain, cleavage of Aβ via the amyloidogenic pathway, and abnormal tau phosphorylation (Durazzo et al., 2014); in addition to being a contributor to vascular dysfunction, long-term disability, and harming several organs (Archie et al., 2022). Cigarette smoking is known to impair nitrergic nerve function and nitric oxide synthesis in cerebral vascular endothelial cells, which interferes with blood flow and glucose metabolism in the brain, promoting Aβ synthesis (which further impairs blood flow), reducing intraneuronal clearance of Aβ, and potentially leading to impaired cognitive function and AD (Toda and Okamura, 2016). It has been shown that healthy, older individuals with a history of cigarette smoking, have significantly greater brain atrophy rate across several brain regions, in particular those associated with early-stage AD (Durazzo et al., 2012). Another study has reported that cigarette smoking and oxidative stress increase APP and Aβ expression in pulmonary artery smooth muscle cells, which can have implications in the association of lung function decline and cognitive decline (Karoor et al., 2022). Additionally, Aβ_40_ and, especially, Aβ_42_ were found to reduce the levels of contractile proteins SM22 and Calponin (Karoor et al., 2022).

Limited evidence is available regarding the relationship between smoking and CAA, specifically. A study analyzing data from 3 community-based cohort studies found that smokers were at increased risk for CAA (RR = 1.11) (Ma et al., 2025). A cohort study in participants with familial CAA (Dutch-type hereditary CAA) found no association between smoking and age at first ICH nor time of ICH recurrence (Voigt et al., 2024).

### 4.7 Alcohol use

Alcohol is known for its acute effects on the brain, with temporary cognitive impairment and behavioral changes being commonly observed (Letenneur, 2004). Although sometimes classified as a risk factor for AD and other dementias, reported effects are conflicting and typically depend on the dose (Wiegmann et al., 2020). According to literature reviews, while light-to-moderate drinking (∼1–3 drinks/day) is associated with lower risk of all-cause and vascular dementia, high alcohol consumption (>14 drinks/week) is associated with increased risk of dementia, reduced brain volume, and brain damage (Letenneur, 2004; Wiegmann et al., 2020). For example, studies have shown that moderate wine consumption (<1–2 glasses/day) is associated with lower risk of AD (Orgogozo et al., 1997; Lemeshow et al., 1998). Meanwhile, a study that looked at 397 dementia cases over a mean follow-up of 23 years, found that dementia risk was higher in people who abstained from alcohol in midlife, as well as in those who consumed over 14 units of alcohol per week (Sabia et al., 2018). In contrast, data from the ALBION study showed that even light-to-moderate alcohol intake was associated with higher Aβ deposition, and Tau/Aβ and pTau/Aβ positivity in healthy individuals, compared to those who abstained from alcohol (Drouka et al., 2025). Several studies in rodent models of AD report that ethanol consumption exacerbates cognitive-behavioral deficits and amyloid pathology and disrupts metabolism and excitatory/inhibitory balance in the brain (Huang et al., 2018; Hoffman et al., 2019; Day et al., 2023). However, in line with human data, preclinical data also supports that there may be a dose-dependent relationship between alcohol consumption and AD/dementia-relevant pathology (Lundgaard et al., 2018; Kang et al., 2024).

Data on the relationship between alcohol use and CAA, specifically, are far rarer. A study analyzing data from 3 community-based cohort studies found no significant relationship between alcohol consumption and CAA (Ma et al., 2025). Additionally, a cohort study in participants with familial CAA (Dutch-type hereditary CAA) found no association between alcohol use and age at first ICH nor time of ICH recurrence (Voigt et al., 2024).

Due to studies having different definitions of what constitutes one drink; what differentiates light, from moderate, from heavy drinking; and many times not differentiating between gender; drawing conclusions about the effects of alcohol consumptions on dementia risk, or CAA more specifically, is complicated (Wiegmann et al., 2020).

### 4.8 Traumatic brain injury (TBI)

Traumatic brain injury is a condition in which normal brain function is impaired by an external force. TBI increases the risk for cognitive decline and dementia and accelerates the age of onset (Shively et al., 2012; Li et al., 2016). It has been shown that patients with TBI may have accelerated onset of cognitive decline by 2+ years (Li et al., 2016) and that, although dementia risk decreases over time, it is still evident for over 30 years post trauma, especially in cases of severe TBI and of repeated TBIs. A retrospective cohort study conducted from 2005 to 2011 found that patients who were 55+ years old with moderate-to-severe TBI, or patients who were 65+ years old with mild TBI, were at increased risk of developing dementia (Gardner et al., 2014). TBI that occurs in early to midlife is associated with 2–4 times higher risk of dementia later in life, and even higher in the case of multiple TBIs (Shively et al., 2012; Ramalho and Castillo, 2015). Of note, long-term cognitive impairment following TBI episode(s) can still occur despite normal-looking MRIs and CT scans (Ramalho and Castillo, 2015).

Pathophysiological mechanisms that occur following TBI that may contribute to dementia risk are complex, and brain injuries are highly heterogeneous due to variation in force, rotation, number of injuries sustained, and other extenuating factors. Multiple case studies have reported the development of early-onset CAA in individuals <55 years of age with a history of moderate-to-severe TBI, presenting with ICH (Ehling et al., 2012; Purrucker et al., 2013; Nakayama et al., 2017; Oblak et al., 2022). However, a valid point made by Banerjee and Werring (2022) is that these cases of CAA following TBI may not be due to the injury itself, but rather result from neurosurgery performed to treat the TBI. These authors highlighted that neurosurgery, often involving cadaveric materials or spread by contaminated surgical instruments, can cause iatrogenic CAA, due to Aβ acting as a proteopathic seed.

Observational studies suggest that repeated mild TBI may also impact the development of CAA. A study of 357 contact athletes vs. a community-based cohort reported that contact sport participation was associated with more CAA (Standring et al., 2019). CAA was also associated with chronic traumatic encephalopathy (CTE), suggesting there may be some interaction between these neuropathologies (Standring et al., 2019). In a post-mortem study of retired male professional soccer players with dementia, moderate CAA was found in 4/6 and severe CAA in 1/6 cases (Ling et al., 2017). Taken together, these findings suggest that TBI may influence the initiation and/or progression of CAA. Experimental evidence is lacking in animal models of CAA; however, in AD models, TBI was shown to increase Aβ deposition, worsen cognition, delay glial activation, and enhance expression of inflammatory cytokines (Breunig et al., 2013; Kokiko-Cochran et al., 2016; Shishido et al., 2016; Zyśk et al., 2019).

## 5 Drug repurposing for CAA treatment and/or prevention

Given the prevalence and clinical impact of CAA, both as a standalone condition and in combination with AD, the timely identification of novel treatment strategies is critical. Drug repurposing, also known as drug repositioning, is the process of identifying new therapeutic uses for existing drugs that are already approved for other conditions or that have passed significant stages of development. Repurposing drugs can reduce the time required for developing, testing, and approving a new drug. Additionally, the knowledge of the drug profile (e.g., dosing, safety, tolerability, adverse effects) results in faster and less expensive development programs (Cummings et al., 2025). However, drug repurposing for CAA presents challenges, including uncertainty about whether doses effective for the original indication are appropriate for the new use, potential drug-drug interactions, and issues related to BBB penetration capability (Cummings et al., 2025). A summary of repurposed drugs can be found on Table 5.

**TABLE 5:** Repurposed drugs for cerebral amyloid angiopathy (CAA) management.

Drug	Original indication	Proposed mechanism in CAA	Level of evidence	Clinical trial status
Telmisartan (Motzko Noto et al., 2025)	Cardiovascular disease; hypertension [angiotensin receptor blocker (ARB)]	Blocks brain AT_1_ receptors, preventing angiotensin II binding; therefore reducing neuroinflammation and oxidative stress, enhancing vascular function, and protecting neurological function.	Low • Does not attenuate CAA in Tg-SwDI mice. Modest benefits for cognitive function, vascular density, and neuroinflammation.	Clinical trials needed
Lisinopril (Motzko Noto et al., 2025)	Cardiovascular disease; hypertension [angiotensin-converting enzyme (ACE) inhibitor]	Limits the production of angiotensin II and its subsequent binding to AT1R; therefore reducing neuroinflammation and oxidative stress, enhancing vascular function, and protecting neurological function.	Low • Does not attenuate CAA in Tg-SwDI mice. Modest benefits for cognitive function, vascular density, and neuroinflammation.	Clinical trials needed
Perindopril (Arima et al., 2010)	Cardiovascular disease; hypertension [angiotensin-converting enzyme (ACE) inhibitor]	Limits the production of angiotensin II and its subsequent binding to AT1R; therefore reducing neuroinflammation and oxidative stress, enhancing vascular function, and protecting neurological function.	Low • Reduces recurrence of CAA-related ICH in humans.	PROGRESS trial complete (2010)
Cilostazol (Maki et al., 2014; Saito et al., 2016, 2023; Chien et al., 2023)	Reduce intermittent claudication symptoms [phosphodiesterase inhibitor]	Proposed to aid in the clearance of Aβ via enhancement of perivascular drainage of interstitial fluid.	Low • Reduces Aβ_40_ deposits and ameliorates cognitive impairment in Tg-SwDI mice. • Did not prevent cognitive decline in clinical trial.	Phase 2 clinical trial in patients with mild cognitive impairment (COMCID trial) complete (2020)
Minocycline (Fan et al., 2007b; Yan et al., 2015; Voigt et al., 2023; Bax et al., 2024)	Prevention/treatment of bacterial infections [tetracycline antibiotic]	Anti-inflammatory and neuroprotective properties. Inhibits the degradation of the perivascular extracellular matrix; may help to prevent hemorrhagic events.	Low • Reduces number of microhemorrhages, activated microglia, and IL-6 levels in Tg2576 mice. • Improves behavioral performance in Tg-SwDI mice. • Reduces ICH recurrence in humans.	• Single-center cohort study in humans (2024) • BATMAN clinical trial (unknown status)
Metformin (Inoue et al., 2021; Teng et al., 2021)	Type 2 diabetes mellitus [biguanide]	Activation of AMP-activated protein kinase (AMPK), potentially contributing to reduced inflammation, decreased oxidative stress, and enhanced cellular repair processes	Low • Reduces cerebrovascular deposits of Aβ in the cortex and hippocampus; increases expression of insulin-degrading enzyme in the hippocampus of mice. • Improves cognitive outcomes and lowers cerebral small vessel disease burden in humans.	Clinical trials needed
Memantine (Reisberg et al., 2003; Inoue et al., 2019; Tang et al., 2023)	Symptomatic treatment of moderate to severe AD [N-methyl-D-aspartate (NMDA) receptor antagonist]	Increases the expression of insulin-degrading enzyme (IDE), which breaks down Aβ	Low • Reduces levels of Aβ_40_, parenchymal plaques, active astroglia and microglia; improves spatial working memory in APP23 mice.	Clinical trials needed

As discussed previously, strict blood pressure control is a cornerstone of managing CAA to reduce the risk of recurrent ICH. There are several classes of antihypertensive drugs that lower blood pressure through different mechanisms, including agents that target the renin-angiotensin-aldosterone system [Angiotensin-Converting Enzyme Inhibitors (ACEIs) and Angiotensin II Receptor Blockers (ARBs)], calcium channel blockers, thiazide diuretics, and beta-blockers, among others. Of note, observational studies have indicated that certain antihypertensive drugs, particularly centrally-acting ARBs and ACEIs, are associated with a reduced risk of all-cause dementia, AD, and VCID specifically (Takeda et al., 2009; Li et al., 2010; Chiu et al., 2014; Ding et al., 2020; den Brok et al., 2021; Deng et al., 2022; Lundin et al., 2024). Findings suggest potential neuroprotective effects beyond blood pressure regulation due to their ability to cross the BBB and influence the brain RAS, reduce neuroinflammation, protect neurological function, protect against oxidative stress and reduced blood flow, and enhance neurovascular coupling (Saavedra, 2012; Glodzik and Santisteban, 2021; Zhou et al., 2024).

A recent study by our group found that treatment with sub-depressor doses of telmisartan (an ARB) or lisinopril (an ACEI) from ∼3 to 8 months of age partially preserved cognitive-behavioral functions in Tg-SwDI mice. Specifically, telmisartan rescued object recognition memory, while both telmisartan and lisinopril preserved spatial memory in the Barnes maze (Motzko Noto et al., 2025). However, no reductions in Aβ levels were observed, and only limited improvements in vascular density and neuroinflammation were detected (Motzko Noto et al., 2025). Additionally, Perindopril, a centrally active ACE inhibitor, was shown to reduce by 77% the recurrence of CAA-related ICH in humans (Arima et al., 2010). These findings support the hypothesis that certain antihypertensive drugs may protect against CAA, independent of their blood pressure-lowering effects (Motzko Noto et al., 2025), although clinical trials are necessary to further explore this potential.

Cilostazol is a selective type 3 phosphodiesterase inhibitor used to reduce symptoms of intermittent claudication by increasing levels of cyclic adenosine monophosphate (cAMP) and, in turn, inhibiting platelet aggregation and improving blood flow to the legs. Despite clinical advice that antiplatelet therapy should not be used in patients with CAA, it was shown that cilostazol treatment reduced Aβ_40_ deposits and ameliorated cognitive impairment in Tg-SwDI mice (Maki et al., 2014) and preserved cognitive function in AD patients with peripheral arterial occlusive disease (Chien et al., 2023). Although cilostazol is an antiplatelet agent, authors from the study in Tg-SwDI mice suggested that the benefits of cilostazol were likely mediated by its vasculotropic effects rather than its antiplatelet effects, as chronic aspirin treatment did not affect cerebrovascular Aβ accumulation or cognitive function in this CAA mouse model (Maki et al., 2014).

Minocycline, an antibiotic with anti-inflammatory properties, reduced the number of microhemorrhages in the brains of Tg2576 mice (Yan et al., 2015). In 12-months-old Tg-SwDI mice treated every other day for 4 weeks with minocycline, the drug significantly reduced the number of activated microglia and the levels of IL-6, and improved behavioral performance, despite no changes in the accumulation and distribution of Aβ, compared to saline-treated Tg-SwDI mice (Fan et al., 2007b). Of note, minocycline treatment appeared safe and well tolerated in a small group of patients with aggressive CAA, also reducing ICH recurrence (Bax et al., 2024).

Several studies are looking into the potentially neuroprotective, disease-modifying effects of anti-diabetic drugs for the treatment of cognitive decline and dementia. Inoue et al. (2021) suggested that metformin, a first-line T2DM drug, could attenuate CAA severity, showing that it significantly reduced cerebrovascular Aβ deposits in the cortex and hippocampus of APP23-*ob/ob* mice (a mixed mouse model of CAA and T2DM), while increasing the expression of insulin-degrading enzyme in the hippocampus. Long-term metformin use in patients with T2DM was associated with improved cognitive outcomes and lower cerebral small vessel disease burden, suggesting that its cognitive benefits may be partly mediated through vascular mechanisms (Teng et al., 2021). However, as the cerebral small vessel disease burden score in this study included several vascular pathologies, including cerebral microbleeds, lacunes, white matter hyperintensities, and perivascular spaces (Teng et al., 2021), further research is needed to clarify metformin’s specific effects on CAA and related cerebrovascular conditions.

Memantine (Namenda^®^) is a non-competitive N-methyl-D-aspartate (NMDA) receptor antagonist that has been used to treat symptoms of moderate to severe AD since 1989 (Reisberg et al., 2003; Tang et al., 2023). In the brain, glutamate is the main excitatory neurotransmitter that exerts its activity through activation of postsynaptic NMDA receptors. NMDA receptor activation is vital for memory processes; however, hyperactivation of these receptors leads to excitotoxicity and enhanced neuronal vulnerability, contributing to AD pathogenesis and dementia (Reisberg et al., 2003; Tang et al., 2023). Memantine, by inhibiting the NMDA receptors, enhances cholinergic signaling and neurogenesis, reduces neuroinflammation, and relieves AD symptoms (e.g., impaired memory and cognitive decline) (Tang et al., 2023). However, despite providing symptomatic relief, memantine does not modify the course of the disease (Tang et al., 2023). In CAA, the effects of memantine have not been as widely studied. Inoue et al. (2019) reported that APP23 mice treated with 30 mg/kg/day memantine from 6 to 18 months of age presented reduced cerebrovascular Aβ in leptomeningeal and cortical lesions; reduced levels of soluble and insoluble Aβ_40_, parenchymal Aβ plaques, active astroglia and microglia in the cerebral cortex and hippocampus; increased levels of hippocampal and vascular insulin-degrading enzyme; and improved spatial working memory. The results suggested that memantine was able to reduce cerebrovascular Aβ deposits by enhancing the expression of Aβ-cleaving insulin-degrading enzymes (Inoue et al., 2019). Further research is needed to determine the appropriateness of memantine in managing CAA-related vascular pathology in humans.

## 6 Conclusions and future directions

Cerebral amyloid angiopathy is a cerebrovascular disorder, highly prevalent in older adults, that contributes to cognitive decline, dementia, and an increased risk of ICH. Despite its clinical significance, there are currently no FDA-approved treatments for CAA. CAA is highly comorbid with AD, exacerbating AD pathology and clinical manifestations. The presence of CAA in AD patients also complicates treatment with recently approved monoclonal antibodies, as it increases the risk of ARIA, including vasogenic edema and hemorrhage. Therefore, sensitive and specific fluid biomarkers and imaging methods capable of reliably detecting CAA are necessary to identify AD patients at increased risk for ARIA. Given the current lack of effective treatments, there is an urgent need to develop new therapeutic approaches appropriate for treating CAA as a standalone condition or when mixed with AD, including those that reduce Aβ accumulation and inflammation, promote vascular and neuronal health, and prevent hemorrhages. While some repurposed drugs have demonstrated potential, their efficacy still requires validation in clinical trials. Positive lifestyle changes (e.g., vascular risk reduction, diet, exercise) have also shown promise for slowing CAA progression and mitigating associated risks, warranting further investigation.

Despite the extensive research done on CAA, several gaps still exist. Although the cause of sCAA is believed to be through impaired perivascular clearance, the exact mechanism or reason as to why this happens requires further investigation. Underrepresentation of non-white populations in CAA studies results in non-representative results, making it hard to generalize risk factors to evaluate potential therapeutics. Currently available diagnostic techniques are invasive and lack accuracy, making the accurate diagnosis of CAA only possible post-mortem. Additionally, related complications that can arise or be exacerbated by CAA (e.g., AD, ICH, inflammation) need to be better understood in order to develop safe and efficacious treatments.

Future studies should focus on understanding the cause and progression of the disease, to develop better diagnostic techniques and treatments. This should include a combination of epidemiological studies, laboratory tests, and brain imaging. Having a better understanding of CAA etiology would aid in the development of strategies to improve the removal of Aβ from cerebral vessels, as well as finding potential new treatments to protect the integrity of the cerebral vasculature from rupture. Additionally, exploring potential biomarkers in the blood and the CSF would provide the means for early diagnosis, as well as better monitoring of treatment effects. However, because definitive CAA diagnosis can only be given postmortem, these studies could take several years to be completed, pressing for a more immediate approach. Additionally, due to the high comorbidity between CAA and AD, studies should consider the increased risk for ARIA carried by certain treatments, particularly anti-amyloid therapies. Future studies on the subject should consider monitoring patients for ARIA-E and ARIA-H frequently through MRIs and by paying close attention to potential symptoms such as headache, confusion, and seizures. However, due to the elevated risk of iatrogenic ARIA, other alternatives for treatment should be considered.

The ability of currently approved drugs to be repurposed for the treatment of CAA should be further investigated, as it could greatly expedite development programs and treatment approval, in addition to reducing the cost of treatment. Although several drug types have shown potential therapeutic benefits for CAA treatment, the disease burden and lack of FDA-approved drugs make the need for alternatives urgent. Future studies could focus on developing clinical trials to assess the safety and efficacy of some of the drugs mentioned in this review that have shown preclinical and clinical evidence supporting their efficacy, in addition to clinical trials to explore the effects of drugs with supported evidence of effectiveness in other cerebral vascular diseases and AD. For example, drugs like GLP-1 agonists, anti-hypertensives, and anti-cancer agents have all shown positive effects on AD and cognitive decline (Ancidoni et al., 2021; Kehoe et al., 2021; Fessel, 2024), but their effects on CAA have not yet been deeply explored.

However, in order to consider repurposing a drug, drug profiles need to be well understood, as well as their effect on different CAA subtypes and disease stage. Additionally, individual patient factors should be considered, including sex/gender, age, ethnic group, genetic factors (e.g., APOE status), vascular risk factors, etc. To this day, some groups are greatly underrepresented in research, despite being highly affected by the disease. For example, African Americans, Hispanics and Latinos, Asian Americans, as well as individuals with disability, and individuals from lower socioeconomic backgrounds and from the LGBTQIA + community, are often not included in dementia studies/clinical trials. Additionally, preclinical studies often do not include females, limiting the generality of the observations. Going forward, studies should integrate representants of all groups, without which results cannot, and should not, be generalized to the entire population.

Additionally, increasing awareness of the importance of healthy lifestyles beginning at childhood and extending throughout the lifespan should greatly reduce the incidence of CAA precluding the need for treatment. Educating people on the subject through credible sources, workshops, and community initiatives could greatly spread the information and positively impact those who might not have access to more expensive treatments and alternatives. Additionally, strategies should be developed to motivate individuals to engage in healthy lifestyles.

Considering the several risk factors of CAA and the multifactorial nature of the disease, multi-domain interventions should be considered. Such interventions combine lifestyle adjustments with therapeutic interventions, addressing several risk factors and characteristics of the disease at once, possibly improving the results obtained. The characteristics and lifestyle of each patient should be considered in order to tailor the treatment plan and increase the likelihood of success. A similar approach was taken for dementia with the Finnish Geriatric Intervention Study to Prevent Cognitive Impairment and Disability (FINGER) trial, in which participants (60–77 years of age) were randomized to multidomain intervention [dietary guidance, exercise, cognitive training, social activity, and monitoring of cardiovascular health (blood pressure, cholesterol, blood glucose, obesity)], which lasted for 2 years, and to a control group that received regular health advice. Results found a 25% improvement in overall cognition, 83% in executive function, 40% in complex memory tasks, and 150% in processing speed (Ngandu et al., 2015). Future studies could explore if these results remain true with CAA specifically, in order to determine the benefit of a multi-domain approach.

The need for further investigation of CAA, for new clinical trials, and for spreading awareness is pressing, as they could advance the research on this disease, increasing the chances of finding a cure or, on the very least, a treatment to mitigate a condition that currently affects many people, causing physical, emotional, social, and financial burdens.

## Footnotes

1
https://clinicaltrials.gov/

2
https://www.cerebrolysin.com/

3
https://www.alnylam.com/sites/default/files/pdfs/mivelsiran-aln-app-fact-sheet.pdf

4
https://www.who.int/news-room/fact-sheets/detail/hypertension

5
https://aaic.alz.org/downloads2025/USPOINTERALZNewsRelease.pdf

6
https://www.alz.org/us-pointer/study-overview.asp

7
https://www.alz.org/us-pointer/study-results.asp
